# Synthesis and Characterization of Glyco-SAMs on Gold Nanoparticles: A Modular Approach Towards Glycan-Based Recognition Studies

**DOI:** 10.3390/molecules30183765

**Published:** 2025-09-16

**Authors:** Sebastian Kopitzki, Joachim Thiem

**Affiliations:** Department of Chemistry, Institute of Organic Chemistry, University of Hamburg, Martin-Luther-King-Platz 6, D-20146 Hamburg, Germany; sebastiankopitzki@web.de

**Keywords:** carbohydrates, synthesis, self-assembly, monolayers, molecular recognition

## Abstract

Within recent years, the interest in tools to investigate carbohydrate-protein (CPI) and carbohydrate-carbohydrate interactions (CCI) has increased significantly. For the investigation of CPI and CCI, several techniques employing different linking methods are available. For mimicking the glycocalyx self-assembled monolayer (SAM) formation of carbohydrate derivatives on gold nanoparticles is most appropriate. In contrast to methods for glyco-SAM formation used previously to analyze CPI/CCI, the novel approach allows for a facile and rapid synthesis to link spacers and carbohydrate derivatives and enhances the binding event by controlling the amount and orientation of ligand. For immobilization on biorepulsive aminooxy functionalized gold nanoparticles by oxime coupling, diverse aldehyde-functionalized glycan structures of mono-, di-, and complex trisaccharides were synthesized, employing several facile steps including olefin metathesis. Effective immobilization and first binding studies are presented for the lectin concanavalin A. This novel and advantageous immobilization method is presently employed in various biomimetic studies of carbohydrates and carbohydrate-based array development for diagnostics and screening.

## 1. Introduction

Eukaryotic cell membranes are coated by a so-called “glycocalix” consisting of a complex mixture of glycoproteins, glycolipids, complex oligosaccharides, glycoconjugates, and proteoglycans with a size up to 100 nm. Cellular processes, such as bacterial and viral infection, cancer metastasis, modulation and activation of the immune system, tissue differentiation and development, and further intercellular recognition events are largely controlled on the molecular level by these glycoconjugates [1,2,3]. Cellular recognition events involving carbohydrate derivatives are carbohydrate-protein-interactions (CPI) between sugars and lectins [4] or selectins [5], as well as carbohydrate-carbohydrate-interactions (CCI) [6]. Whereas antigen-antibody interactions range from 10^−8^ to 10^−12^ M, CPI and CCI are typically weak in in vitro testing, with K_D_ values in the millimolar or high micromolar range [7,8,9]. On the other hand, multivalent presentation of carbohydrate recognition units can increase binding affinity considerably [10,11]. Despite intensive studies of the glycocalyx by glycobiology, to date, there is no general mechanistic concept of carbohydrate recognition [12,13,14,15,16,17].

In order to facilitate studies of carbohydrate-based recognition events, new analytical and synthetic approaches are of interest. These include chemoenzymatic and automated solid phase syntheses of oligosaccharides, and glycomimetics as well as chemical tools, microarrays, glyconanoparticle technology, and molecular modeling [18,19,20,21]. Particular advantageous for mimicking the glycocalyx are the self-assembly of thiol-functionalized molecules on gold surfaces, first introduced by Whitesides et al. [22]. The conception of glyco-SAMs proved to be particularly well suited for the investigation of molecular interactions of carbohydrates, allowing characterization as well as control of density and orientation of carbohydrate ligands [23,24]. Furthermore, advantageous studies of glyco-SAMs on gold employed spectroscopic techniques like surface plasmon resonance, ellipsometry, atomic force microscopy, or X-ray photoelectron spectroscopy [25,26,27].

Gold nanoparticles represent important carrier and delivery materials for biological active compounds in nanomedicine, since they provide both multivalency and multifunctionality. Therefore, their preparation, toxicity, and application for in vivo imaging, diagnosis, and therapy are intensively discussed [28]. Colloidal gold was used for therapeutic and cosmetic properties at an early stage by the Chinese and Egyptians. The first scientific publication about gold nanoparticles (AuNP) by Faraday in 1857 reported and discussed the reason for the intensive red colour of colloidal gold [29]. Employing Maxwell’s electromagnetic equation, the visual light absorption of AuNP was described by Mie in 1908 [30]. Nanoscaled AuNPs are typically available in sizes from 1 nm to 120 nm, in a size range of proteins and other biomacromolecules, and show plasmon band absorption. Further, in many other physical properties, the colloidal form differs from the metallic bulk form of gold. These changed properties of AuNP are of interest for optics, catalysis, and material science, but also for biology, biochemistry, and medicine [31]. There are reports about the functionalization of AuNP with proteins, DNA, and RNA [32,33], and increasingly examples in the carbohydrates field are published with AuNP as ideal platforms to mimic the glycocalyx of cell membranes [34,35,36,37].

Au-NP are predominantly synthesized by reduction procedures from so-called “gold acid” (HAuCl_4_). The Turkevitch protocol, introduced already in 1951, is widely used for the preparation of various nanoparticle constructs [38]. Here, an aqueous solution of the Au^III^ salt is reduced by sodium citrate to obtain Au^0^ colloids [39]. Subsequent ligand exchange reaction with thiols or other thio derivatives on the citrate-stabilised Au-NP provides the individual functionalized Au-NP [40,41]. This ligand exchange results in an elevated stability of Au-NP due to the strength of the Au-S bond (ca. 50 kcal/mol) [42]. Recent developments of the Turkevitch protocol improved the control of size and distribution, thus nowadays monodisperse Au-NP with a controllable size of 9–120 nm can be synthesized [43]. Schiffrin and Brust presented a one-step procedure for thiolated Au-NP. In a phase transfer system of water and toluene, HAuCl_4_ was reduced with NaBH_4_ in the present of alkylthiols and tetrabutylammonium bromide [44]. The sizes of the Au-NP strongly dependant on the utilized alkylthiol, however, this method allows AuNP smaller than 9 nm to be synthesized. To obtain uniform Au-NP with different functionalization, it is necessary to apply a second synthetic step, mostly an organochemical reaction or a thiol ligand exchange according to [45]

(RS)*_n_*AuNP + *m* R’SH → (RS)*_n-m_*(R’S)*_m_*AuNP + *n* RSH

Carbohydrate—functionalized Au-NP were utilized by Penades et al., whose syntheses followed the Brust protocol [46]. Jensen et al. employed syntheses by a modular conception with thiol ligand exchange on citrate-stabilized Au-NP [47]. This modular carbohydrate functionalization of Au-NP was advantageously used throughout this study since citrate-stabilized Au-NP had only to be prepared once and thus allowed for access to many different Au-NP having uniform material, which improves the interpretation of the SPR experiments. 

**Figure 1:**
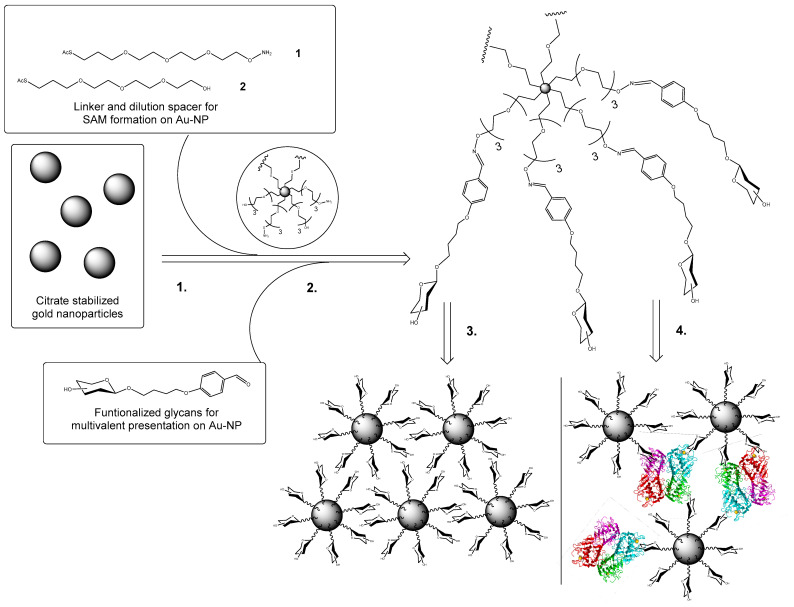
Schematic depiction of the modular approach for the preparation of glyco-SAMs on gold nanoparticles: 1. Pre-synthesized, well-defined gold nanoparticles are used for SAM formation with spacer molecules **1** (attachment) and **2** (dilution), 2. Attachment of carbohydrates and application either 3. in carbohydrate-carbohydrate interactions or 4. in carbohydrate-protein interactions.

The novel modular approach for immobilization of synthetic glycosides on gold nanoparticle reported herein is based on (1) the control of particles size by bulk preparation of goldnanoparticles by the Tuchevitch protocol, (2) control of ligand density by non-specific binding via tetraethylene glycol tethers, (3) the control of ligand density via tether length, provision of functionalization by preparation of mixed SAMs in different ratio, (4) a facile in situ immobilization by thioalkanes on gold as SAMs and (5) a facile in situ attachment of glycoderivates by reductive amination (Figure 1). 

For control of ligand interdistance, benzaldehyde-functionalized glycoderivatives were employed, in which the carbohydrate head groups were attached to amine-bearing SAMs and diluted with biorepulsive spacers. These biorepulsive compounds essentially require a long alkane chain and a terminal oligoethylene glycol moiety for self-assembly. The alkane chain is needed to form strong van der Waals interactions between spacers, to promote accurate SAM formation [48], and the oligoethylene glycol moiety is employed to exclude non-specific adhesion of biomaterial to the SAM [49]. In addition, by insertion of the amino function, an anchor group is provided.

Whereas conventional glycosylations usually lead to anomeric mixtures and thus require tedious chromatographic separations, the present approach for covalent immobilization via reductive amination of the aldehyde-linking partner facily gives uniform components. Thus, the aldehyde functions are easily introduced to anomerically pure allyl glycosides by cross-metathesis [50,51]. In contrast to ring closing metathesis (RCM) and ring opening metathesis polymerization, cross metathesis (CM), especially in carbohydrate chemistry, is less developed [52]. Based on recent reports, which solved the problems of self-metathesis and could also use aqueous CM [53,54], olefin metathesis was applied to modify carbohydrate derivatives. In fact, synthesis of glyco-SAMs could be successfully accomplished by incubation of plain gold sensor surfaces with amino-functionalized and biorepulsive spacers, following subsequent attachment of glycoderivatives by reductive amination. Evidence of effective immobilization of carbohydrate components could be demonstrated via initial binding experiments of the lectin concanavalin A to modified SAMs carrying α-mannopyranoside structures.

**Figure 2:**
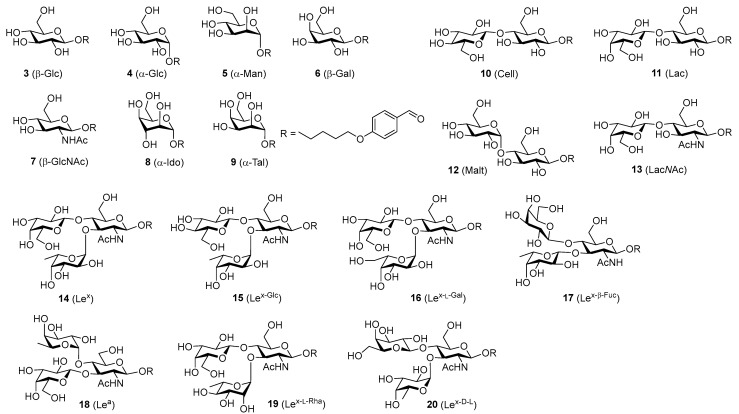
Benzaldehyde functionalized glycosides used for the oxime formation on gold nanoparticles. Abbreviated names of carbohydrate head groups: **14** (Le^x^): Galα(1–4)[Fucα(1–3)]Glc*N*Ac; **15** (Le^x-Glc^): Glcβ(1–4)[Fuc56(1–3)]Glc*N*Ac; **16** (Le^x-L-Gal^): Galβ(1–4)[L-Galα(1–3)]Glc*N*Ac; **17** (Le^x-β-Fuc^): Galβ(1–4)[Fucβ(1–3)]Glc*N*Ac; **18** (Le^a^): Galβ(1–3)[Fucα(1–4)]Glc*N*Ac; **19** (Le^x-L-Rha^): Galβ(1–4)[Rhaα(1–3)]Glc*N*Ac; **20** (Le^x-D-L^): L-Galβ(1–4)[D-Fucα(1–3)]Glc*N*Ac.

For the present study eighteen benzaldehyde-functionalized glycan structures were employed. These comprise five monosaccharide derivatives **3**–**9**, four disaccharide derivatives **10**–**13**, and seven trisaccharide derivatives **14**–**20** as depicted in Figure 2 (Figure 2).

## 2. Results and Discussion

### 2.1. Synthesis of Benzaldehyde-Functionalized Carbohydrates

**Scheme 1:**
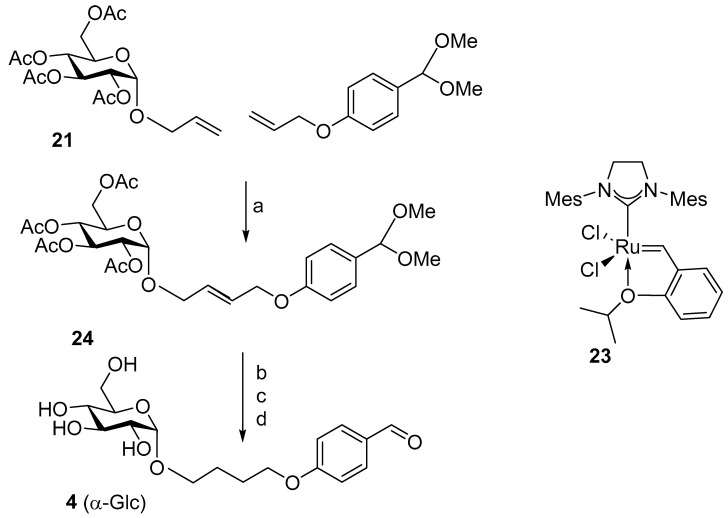
Synthesis of aldehyde-functionalized α-d-glucose. Reagents and conditions: (a) catalyst **23** (10 mol%), **22** (5 eq), 40 °C, 3 h, 91%; (b) H_2_/Pd, PhSPh, EtOAc, RT, 12 h, quant; (c) TFA/H_2_O/THF (0.1/9.9/90 *v*/*v*), RT, 6 h, quant.; (d) NaOMe, MeOH, RT, 6 h, 91% (over 3 steps).

According to previously developed procedures [55] for attachment of glycoderivatives to amino-functionalized SAMs, readily accessible allyl glycosides were chosen as starting components. Thus, allyl 2,3,4,6-tetra-*O*-acetyl-α-d-glucopyranoside (**21**) was treated with para-(allyloxy)benzaldehyde dimethyl acetal (**22**) [55] for olefin metathesis under catalysis with Grubbs-Hoveyda second-generation catalyst **23** [56] to give compound **24** in 91% yield. Employing optimized reaction conditions [57], cross metathesis led exclusively to the (*E*)-configured derivative. Next, deacetalization of the benzaldehyde dimethyl acetal **24** followed by hydrogenation catalyzed employing palladium on charcoal poisoned with diphenyl sulfide and subsequent classical Zemplén deprotection gave the target compound **4** in 91% yield over three steps (Scheme 1).

**Scheme 2:**
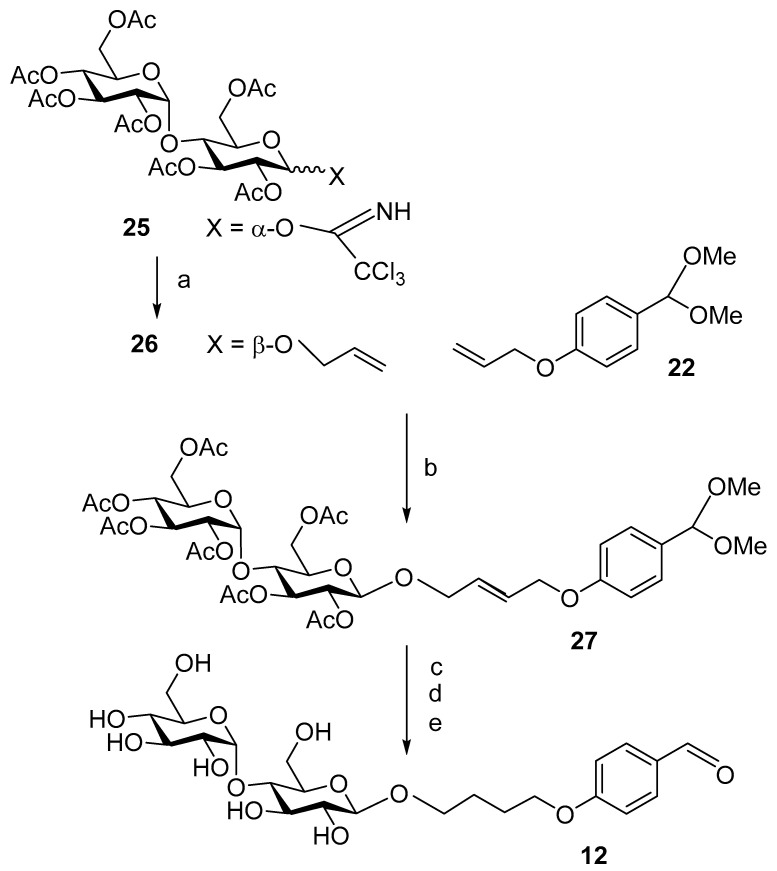
Synthesis of aldehyde-functionalized maltose. Reagents and conditions: (a) AllOH, TSMOTf, 0 °C, 2 h, 83%; (b) catalyst **23** (10 mol%), **22** (5 eq), 40 °C, 3 h, 76%; (c) TFA/H_2_O/THF (0.1/9.9/90 *v*/*v*), RT, 6 h; (d) H_2_/Pd, PhSPh, EtOAc, RT, 12 h; (e) NaOMe, MeOH, RT, 6 h, 77% over 3 steps).

Starting with the α-trichloro acetimidate of peracetylated maltose (**25**) [58], a straightforward glycosylation with allyl alcohol gave the crystalline β-allyl derivative **26** in 83% yield. As before, cross metathesis with **22** under catalysis of **23** resulted in the formation of the (*E*)-component **27** in 76% yield. Finally, employing the above reported three step deprotection method gave the crystalline benzaldehyde-functionalized β-maltose derivative 12 in 77% yield (Scheme 2).

### 2.2. Synthesis of Linker and Dilution Spacers

For the preparation of carbohydrate-functionalized nanoparticles with various ligand densities, both an anchor linker and a dilution spacer are required. Oligoethylene glycol spacers are known to suppress nonspecific binding of biomaterials to surfaces. Therefore, aminoxy tetraethylene glycol linker **1** and the tetraethylene glycol spacer **2** were synthesized by a combination of previously published procedures.

**Scheme 3:**
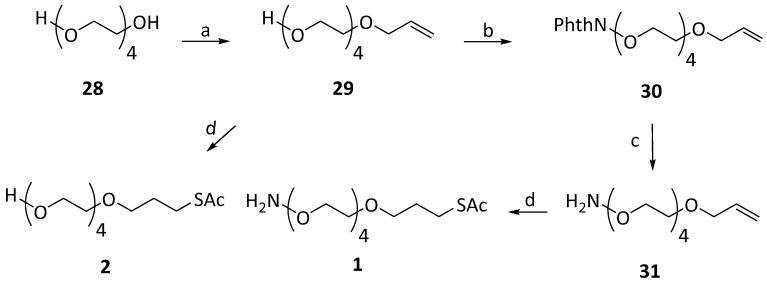
Synthesis of dilution and anchor molecules **1** and **2** for functionalization of Au-NP. Reagents and conditions: (a) NaH, DMF, AllBr, 0°to 20 °C, 12 h, 90%; (b) *N*-hydroxyphthalimide, PS-PPh_3_, DIAD, THF, 20 °C, 16 h, 56%; (c) hydrazine hydrate, MeCN, 20 °C, 2 h, 75%; (d) AcSH, AIBN, THF, hν, 3 h; **1**: quant., **2**: quant.

Mono-*O*-allylation of tetraethylene glycol **28** was realized with NaH and allyl bromide in 90% yield to give compound **29**, the radical thioacetylation of which gave the required dilution spacer component **2** quantitatively. By the Mitsunobu reaction of **29** with *N*-hydroxy phthalimide compound **30** was obtained in moderate yield. Its hydrazinolysis gave **31**, which again, by radical thioacetylation, led to the quantitative formation of the anchor spacer component **1** (Scheme 3).

**Scheme 4:**
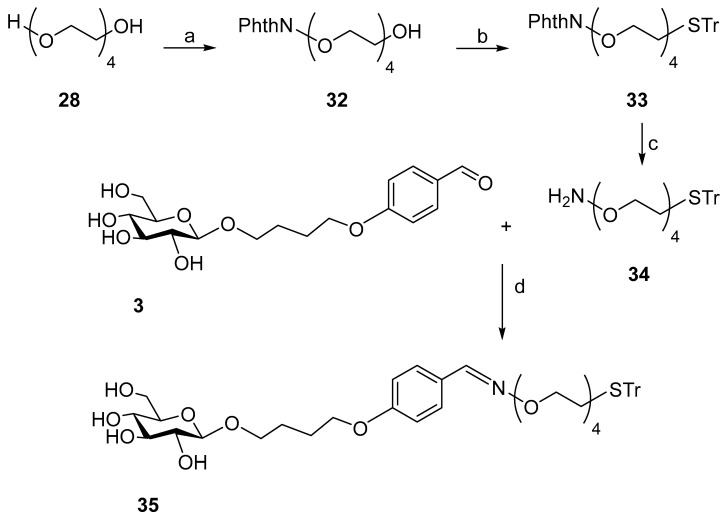
Functionalization of glucose. Reagents and conditions: (a) *N*-hydroxy-phthalimide, PS-PPh_3_, DIAD, THF, 20 °C, 16 h, 37%; (b) TrSH, PS-PPh_3_, DIAD, THF, 0° to 20 °C, 16 h, 78%; (c) hydrazine hydrate, MeCN, 20 °C, 2 h, 97%; (d) **3** (1.1 eq), MeCN, H_2_O, AcOH, 16 h, quant.

For functionalization of saccharides, tetraethylene glycol **28** was treated with *N*-hydroxy phthalimide in a Mitsunobu reaction, employing polymer-bound triphenyl phosphine (PS-Ph_3_P) to facilitate removal of phosphine oxide. The monosubstituted product **32** was obtained in 37%, corresponding to a statistically adjusted [59] yield of 74%. Under corresponding conditions, the next step used again the Mitsunobu method to give the protected thiol **33** (previously reported by another approach [60]) in 78% yield. Hydrazinolysis gave the aminoxy linker **34** in 97% yield, and for exemplary check, this could be coupled with the β-glucopyranoside component **3** to give the oxime **35** quantitatively in an (*E/Z*) ratio of 10:1 (Scheme 4).

**Figure 3:**
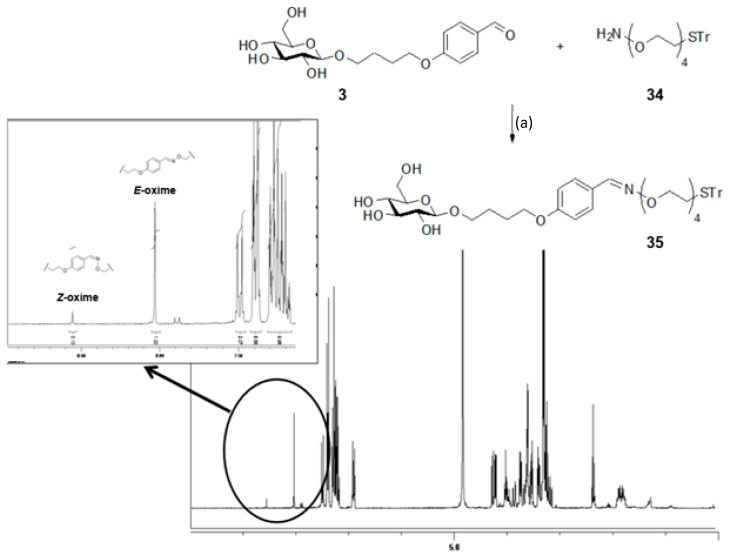
Solution test ligation. Benzaldehyde functionalized glucoside **3** was successfully coupled to solution attachment spacer **34** to give component 35. NMR spectra show that (*E*)-oxime was preferentially formed (*E*/*Z* ratio 10:1). Reagents and conditions: (a) 3 (1.1 eq), MeCN, H_2_O, AcOH, 16 h, quant.

Prior to assembling glycoconjugates on aminooxy functionalized gold nanoparticles, the coupling system was tested in solution. As proven in the synthesis of compound **35** their structures could be assumed to be preferentially (*E*)-oximes, which is relevant for CPI and CCI studies with uniform materials (Figure 3).

### 2.3. Synthesis of Glyconanoparticles

For the binding of free oligosaccharides at their reductive terminal, Jensen et al. [55] employed amino-oxy tetraethylene glycol **34** to give glycoconjugates, which in turn, by their thiol group, formed functionalized Au-NP useful for studies of biological recognition processes. A corresponding approach was to be applied with the novel benzaldehyde-functionalized carbohydrate structures [61,62].

However, to ensure almost homogeneous particle core size for all functionalized gold nanoparticles, a two-step protocol was applied. First, gold nanoparticle formation follows the Turkevitch protocol with subsequent SAM formation using an anchor and dilution spacer. After refluxing HAuCl_4_ in degassed water for 10 min, a 60 °C warm aqueous solution of sodium citrate was added [63] to give Au-NP with diameters of about 13 nm [64,65].

In this work, the functionalization of AuNP by SAM formation was performed according to a protocol by Jensen et al. [66,67,68]. Four types of AuNP (AuNP-1–AuNP-4) were synthesized, in which aqueous solutions of citrate-stabilized AuNP were incubated with methanolic solutions of anchor spacer **1** and diluent spacer **2** in different ratios for 16 h (Table 1).

The functionalized Au-NP were transformed by formation of oximes with benzaldehyde glycoconjugates (Figure 2) employing seven monosaccharide derivatives **3**, **5–6** [55], **4**, **8–9** [69], four disaccharide derivatives **10–11**, **13** [55], **12**, and seven trisaccharide derivatives **14–20** [70] into glyconano particles **GNP-1**–**GNP-19**. Incubation of the Au-NP solution was at pH 4.7 and 40 °C with the corresponding solution of the glycan for 16 h [67].

### 2.4. Characterization of GNPs

**Figure 4:**
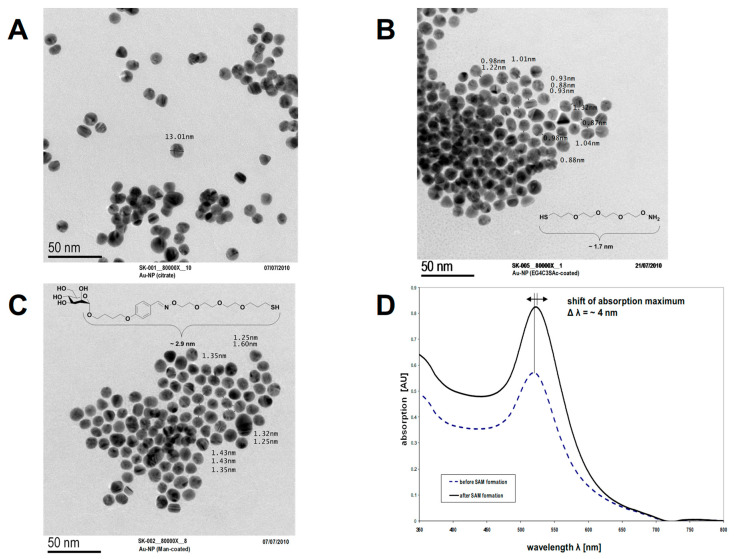
Characterization of glyconanoparticles. (**A**) TEM image of citrate-stabilized gold nanoparticles, average diameter ~13 nm; (**B**) TEM image of **AuNP-1**, average inter particle distance ~1 nm; (**C**) TEM image of **GNP-3**, average inter particle distance ~1.4 nm; (**D**) UV-Vis spectra of gold nanoparticles before (Δλ_SP,max_ = 519 nm) and after (Δλ_SP,max_ = 523 nm) SAM formation with anchor spacer **1**.

Characterization of GNPs was carried out by TEM and quantification of carbohydrate ligands on their surface. The size of the AuNP of 13 nm determined by UV-Vis spectroscopy was confirmed by TEM images and their evaluation. The measurement of a total of 168 particles resulted in a diameter of 13.3 ± 1.3 nm (Figure 4A). Furthermore, in the case of aminooxy-terminated core-shell gold nanoparticles AuNP-1 and mannose GNP (GNP-3), successful functionalizations were confirmed by TEM images (Figure 4B,C). As expected, the SPR absorption of AuNPs changes after SAM formation employing anchor and/or dilution spacers, and that can also be observed visually (Figure 4D). Both the outside appearance as well as the position of their SPR absorption maximum were identical before and after oxime formation.

Quite accurate quantification of carbohydrate ligands per GNP can be performed employing gas chromatography [71] or elemental analysis [72]. However, these methods require tenfold amounts of material in contrast to colorimetric tests [73], employed for determining the number of carbohydrate ligands per GNP [74,75]. Thus, measuring concentrations of carbohydrates by color reactions and subsequent quantification by UV-VIS spectroscopy was of interest. Sulfuric solutions of anthrone and sugars give green products with an adsorption maximum at about 620 nm [76,77], sufficiently distant from the SPR maximum of the AUNP to exclude any interactions during UV-VIS measurements. The number of carbohydrate ligands per GNP results from the quantity of *n*_sugar_ per quantity of *n*_GNP_. The amount of carbohydrate in mg per mg of GNP was determined by an anthrone test. The extinction of the color solution was linear proportional to the amount of sugar as determined by balancing via a linear slope. Detailed information on test procedures and the resulting calculations is described in Section 4, and the resulting number of carbohydrate ligands per GNP is listed in Table 2.

By anthrone tests of the P^k^ antigen trisaccharide [Galα(1–4)Gal(1–3)Glc] attached to AuNP of 13 nm size, Chien et al. observed 820–1300 ligands attached by short or long spacers, respectively [75]. The amount of ligands found in our case is in the same order of magnitude. Employing quantification by the *trim*-(*trim*ellitoyl) system, Jensen et al. measured 440 ligands on AuNP of 13 nm [67]. Lin et al. used AuNP of 20 nm size and established about 650 mannose-C5- and about 880 mannose tetraethylene glycol spacered ligands [78]. The GNPs formed in our experiments clearly showed more replicants per GNP for smaller carbohydrate ligands: that is, for monosaccharide-functionalized GNPs (**GNP-1** to **GNP-6**), there are 1200–1280 ligands per GNP, for disaccharide-functionalized GNPs (**GNP-7** to **GNP-10**), there are 980–1060 ligands per GNP, and for trisaccharide-functionalized GNPs (**GNP-11**, **GNP-14** to **GNP-19**), there are only 880–920 ligands per GNP.

In case of larger carbohydrate structures, a complete allocation of aminoxy functions on AuNP is precluded due to steric reasons. Apparently, this aspect is confirmed, since the number of Le^x^ ligands per GNP does not linearly correlate with the theoretical functionalization of the surface (Table 2, **GNP-11** to **GNP-13**). In the case of **AuNP-3** with a theoretically 33% functionalization of the surface, an amount of 400 ligands was observed. This would correspond to an equivalent of 1200 ligands, assuming a 100% functionalization of the surface for **AuNP-1**; however, 880 ligands were found, corresponding to a deficit of 25%.

### 2.5. Lectin Binding Experiments of GNPs

**Figure 5:**
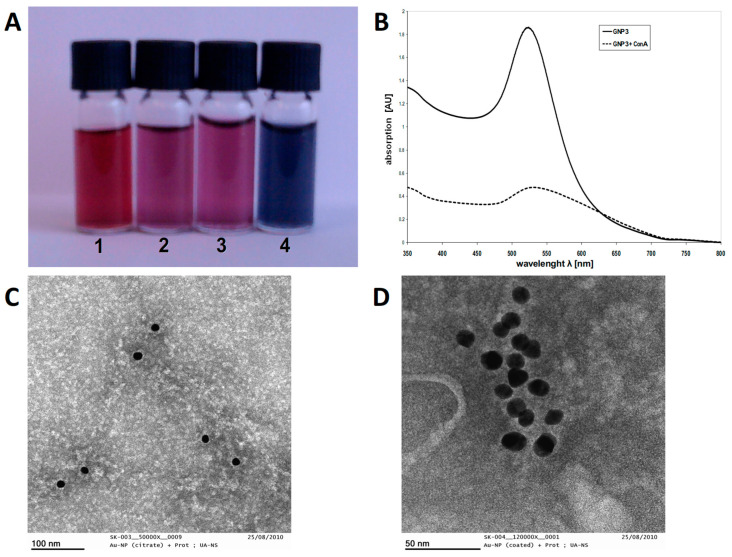
Characterization of ConA binding to mannopyranoside functionalized glyconanoparticle GNP-3. ((**A**)1) citrate stabilized AuNP solution, ((**A**)2) GNP-3 solution, ((**A**)3) GNP-3 solution after incubation with BSA, ((**A**)4) GNP-3 solution after incubation with ConA; (**B**) UV-Vis spectra of GNP-3 before (solid line) and after (dotted line) incubation with ConA; (**C**) *negative stain* TEM image of AuNP-4 after incubation with ConA; (**D**) *negative stain* TEM image of GNP-3 after incubation with ConA.

Assays by UV-Vis as well as TEM experiments could be used to clearly show these novel GNPs to be suited for specific biological recognition processes [79]. Concanavalin A (ConA), the mannose recognizing and binding lectin, can aggregate with mannose by four binding sites [71]. Thus, **GNP-3** (mannose) dissolved in Hepes buffer, showing a normal UV-Vis spectrum with the SPR absorption maximum at about 520 nm, was treated with a Hepes-buffered ConA solution containing Ca^2+^ and Mn^2+^ ions (1 mM each) for 12 h at room temperature. The subsequent aggregation of the GNP was evident by a distinct shift of the absorption maximum and a considerable flattening of the curve (Figure 5B). Further, this ConA-induced aggregation of **GNP-3** could be observed directly, since the solution showed a shift of color from purple to blue (Figure 5A), and after 2 days, all GNP had precipitated.

Furthermore, the aggregation of lectin ConA to the mannose GNP could be substantiated by TEM experiments. By negative sampling, this specific interaction could not be observed. In a conventional TEM image, objects such as AuNP are dark since the electron beam is deflected by the atomic nuclei of heavy elements, and does not hit the CCD camera detector. Thus, organic material made only up from light elements cannot be shown this way; however, by employing the so-called “negative staining method”, for that purpose, the TEM sample is treated with a solution of heavy metals, such as, for instance, phosphotungstic acid, ruthenium tetroxide, or uranyl acetate [80]. This leads to precipitation of heavy metals and dark spots in areas without organic material, and in contrast to light spots in areas with proteins or carbohydrates, etc. [81].

In the negative staining TEM picture of **GNP-3** plus ConA, proteins are observed as light spots (Figure 5D). These bright spots show a diameter of 8 nm, which corresponds to the size of lectin ConA as given by crystal data. For all the **GNP-3,** a systematic arrangement of proteins was observed by an extended light zone around the GNP. This characteristic arrangement for **GNP-3** was visualized by a red circle. However, if **AuNP-4** (SAM only with dilution spacer **2**) was incubated, the light zones around the AuNP were clearly smaller (Figure 5C). Again, in this sample, the lectins are observed; however, not arranged around the AuNP. The light zone around the AuNP of about 1.5 nm is caused by the organic material of the SAMs of the dilution spacer **2**.

By employing a larger magnification, it could be shown that many lectin units are arranged around one **GNP-3** (Figure 5D), and this attachment is shown by a lighter zone around the GNP. Further, a brightening of the GNP itself is observed due to the spheric mannose SAM attached (Figure 5D). Since ConA disposes of four binding sites, several **GNP-3** could be cross-linked. Figure 5D gives an image of how the ConA-linked GNPs are arranged, and some are even piled. Thus, these negative staining TEM studies unequivocally demonstrate these glyconano particles to represent multiple spherical representations of carbohydrate epitopes to be able to incur specific biological bindings. Apparently, the constitution of both GNP and linkers did not show any negative impact on the specificity of the lectin binding. Therefore, these well-characterized GNPs could be employed to perform CCI experiments by SPR measurements.

## 3. Materials and Methods

### 3.1. General

Reagents of commercial quality were purchased from Aldrich (St. Louis, MO, USA), Sigma (St. Louis, MO, USA), or Merck (Darmstadt, Germany) and were used without further purification. Solvents were dried according to standard methods. All reactions were carried out under an argon atmosphere with dry solvents under anhydrous conditions, unless otherwise noted. Analytical thin-layer chromatography (TLC) was performed on precoated aluminum plates (Silica Gel 60F254, Merck5554), compound spots were visualized by UV light (254 nm) and by staining with a yellow solution containing Ce(NH_4_)_2_(NO_3_)_6_ (0.5 g) and (NH_4_)_6_Mo_7_O_24_/4 H_2_O (24.0 g) in 6% H_2_SO_4_ (500 mL) or with 10% H_2_SO_4_ in ethanol followed by heat treatment. For column chromatography, Silica Gel 60, 230e400 mesh, 40e63 mm (Merck) was used. ^1^H NMR and ^13^C NMR spectra were recorded on Bruker (Billerica, MA, USA) AMX-400 (400 MHz for ^1^H, 100.6 MHz for ^13^C) and on Bruker DRX-500 (500 MHz for ^1^H, 125.8 MHz for ^13^C) at 300 K. Chemical shifts were calibrated to solvent residual peaks (CDCl_3_: δ = 7.24 ppm for ^1^H and δ = 77.0 ppm for ^13^C; methanol-d_4_: δ = 3.35 ppm for ^1^H and δ = 49.30 ppm for ^13^C). The signals were assigned by ^1^H-^1^H-COSY, HSQC, HMBC, and, if necessary, NOESY experiments. Hydrogen and carbon atoms are indexed as follows: the sugar residue is numbered as usual from 1 to 6, with the anomeric position being number 1, the atoms of the anomeric spacer moiety then receive numbers with index ‘bu’ for butyl and ‘ar’ for aromatic by consequent numbering starting from the glycosidic bond. Optical rotations were measured using a Krüss Optronic P8000 (589 nm, Hamburg, Germany) at 20 °C. MALDI-TOF-MS was performed on a Bruker Biflex III with dihydroxybenzoic acid or trihydroxyanthracene as matrices in positive reflector mode. FAB-HRMS was performed on a Thermo Finnigan MAT95 XL mass spectrometer (San Jose, CA, USA).

### 3.2. Syntheses

#### 3.2.1. (*E*)-4-[4-(Dimethoxymethyl) Phenoxy] But-2-enyl 2,3,4,6-Tetra-*O*-acetyl-α-d-glucopyranoside (**24**)

Allyl α-d-glucopyranoside tetraacetate (**21**, 200 mg, 515 μmol) and *para*-(allyloxy) benzaldehyde dimethyl acetal (**22**, 1.0 g, 4.80 mmol) [55] were dissolved in dry and degassed dichloromethane (30 mL) and placed into a flame-dried flask containing activated molecular sieves (4 Å) by using standard Schlenk techniques. Grubbs–Hoveyda 2nd generation catalyst (**23**, 33 mg, 52 mmol), dissolved in dry and degassed dichloromethane (1 mL), was added by syringe to obtain a 0.02 m solution. The reaction mixture was heated under reflux for 6 h. Conversion of the starting material was monitored by TLC. The solution was concentrated under reduced pressure, and the crude product was directly purified by column chromatography using silica and a petroleum ether/ethyl acetate gradient (4:1–2:1) to give compound **24** (223 mg, 91%) as a colorless syrup. *R*_f_ = 0.32 (petroleum ether/ethyl acetate, 1:1). [*α*]_D_^20^ = +5.9 (*c* = 1.0 in CHCl_3_). ^1^H-NMR (500 MHz, CDCl_3_) *δ* = 7.32 (d, *J*_Ar_ = 8.4 Hz, 2 H, H-1_arom_), 6.86 (d, *J*_Ar_ = 8.4 Hz, 2 H, H-2_arom_), 5.96–5.80 (m, 2 H, H-2_bu_, H-3_bu_), 5.32 (s, 1 H, H_acetal_), 5.18 (dd, *J*_2,3_ = 2.5, *J*_3,4_ = 2.1 Hz, 1 H, H-3), 5.06 (dd, *J*_3,4_ = 9.6, *J*_4,5_ = 9.9 Hz, 1 H, H-4), 4.99 (dd, *J*_1,2_ = 7.9, *J*_2,3_ = 9.4 Hz, 1 H, H-2), 4.53 (d, *J*_1,2_ = 7.9 Hz, 1 H, H-1), 4.50–4.48 (m, 2 H, H-4_bu_), 4.38–4.32 (m, 1 H, H-1a_bu_), 4.23 (dd, *J*_5,6b_ = 6.4, *J*_6a,6b_ = 11.8 Hz, 1 H, H-6b), 4.15–4.03 (m, 2 H, H-1b_bu_, H-6a), 3.65 (ddd, *J*_4,5_ = 9.9, *J*_5,6a_ = 2.4 *J*_5,6b_ = 4.7 Hz, 1 H, H-5), 3.28 (s, 6 H, OCH_3_), 1.98–2.06 (m, 12 H, 4 H_Ac_); ^13^C-NMR (126 MHz, CDCl_3_) *δ* = 170.5, 170.1, 169.3, 169.2 (4 CH_3_*C*O), 130.6, 128.4, 128.2, 127.9 (4 C_arom_), 114.2 (C-2_bu_), 114.1 (C-3_bu_), 102.9 (C_acetal_), 99.6 (C-1), 72.8 (C-3), 71.7 (C-5), 71.2 (C-2), 68.8 (C-4_bu_), 68.3 (C-4), 67.5 (C-1_bu_), 61.8 (C-6), 52.5 (O*C*H_3_), 20.7, 20.6, 20.5, 20.4 (4 *C*H_3_CO). HRMS (FAB): *calcd.* for C_27_H_37_O_13_ [M + H]^+^ 569.2229, found 569.215.

#### 3.2.2. 4-(4-Formylphenoxy) Butyl α-d-Glucopyranoside (**4**)

Compound **24** (500 mg, 879 μmol) was dissolved in a mixture of THF, water, and TFA (90:9.9:0.1, *v*/*v*) to obtain a 0.2 M solution. After stirring for 1 h, the reaction mixture was diluted with CH_2_Cl_2_ (40 mL), and the reaction was stopped by the addition of triethylamine (4 mL), followed by the addition of water (40 mL). The organic phase was separated and dried with Na_2_SO_4_. The solvent was removed under reduced pressure, and the crude product was used in the next transformation without further purification. For hydrogenation, the aldehyde was dissolved in anhydrous ethyl acetate (20 mL) and placed into a flame-dried flask containing palladium (10% on charcoal) and diphenyl sulfide (0.01 equiv.). The suspension was degassed, and after purging with hydrogen, the mixture was stirred for 12 h. Then the suspension was filtered and thoroughly dried before the residue was redissolved in methanolic sodium methoxide solution (40 mL, 0.1 M). The solution was stirred at room temperature for 6 h, and then the mixture was neutralized with Amberlite IR 120 (H^+^) resin. After filtration and evaporation of the solvent, the crude product was purified by flash chromatography using silica and dichloromethane/methanol (5:1) to give **4** (285 mg, 91%) as a colorless syrup. *R*_f_ = 0.46 (CH_2_Cl_2_/MeOH, 5:1). [*α*]_D_^20^ = +36.0 (*c* = 1.0 in MeOH). ^1^H-NMR (500 MHz, CDCl_3_) δ = 9.81 (s, 1H, H_ald_), 7.83 (d, *J*_Ar_ = 8.7 Hz, 1H, H-1_arom_), 7.00 (d, *J*_Ar_ = 8.7 Hz, 1H, H-2_arom_), 4.12 (t, *J_3bu,4bu_* = 6.7 Hz, 2H, H-4_bu_), 4.09 (d, *J_1,2_* = 3.6 Hz, 1H, H-1), 4.01–3.95 (m, 1H, H-1a_bu_), 3.90–3.83 (m, 1H, H-1b_bu_), 3.55–3.23 (m, 4H, H-3, H-4, H-6ab), 3.20 (dd, *J*_1,2_ = 3.6, *J*_2,3_ = 9.4 Hz, 1H, H-2), 1.96–1.89 (m, 2H, H-3_bu_), 1.83–1.75 (m, 2H, H-2_b_u); ^13^C-NMR (126 MHz, CDCl_3_) δ = 192.0 (C_ald_), 133.1, 116.0 (2 C_arom_), 99.7 (C-1), 77.9 (C-4), 77.7 (C-5), 74.1 (C-2), 71.7 (C-1_bu_), 70.31 (C-4_bu_), 69.9 (C-3), 62.9 (C-6), 27.2 (C-3_bu_), 26.9 (C-2_bu_). HRMS (FAB): *calcd.* for C_17_H_25_O_8_ [M + H]^+^ 357.1544, found 357.1601.

#### 3.2.3. Allyl 2,3,6-Tri-*O*-acetyl-4-*O*-(2,3,4,6-tetra-*O*-acetyl-α-d-glucopyranosyl)-β-d-glucopyranoside (**26**)

A solution of 2,3,6-tri-*O*-acetyl-4-*O*-(2,3,4,6-tetra-*O*-acetyl-α-d-glucopyranosyl)-α-d-glucopyranosyl-trichloroacetimidate (**24**, 781mg, 1.00 mmol) [58] and allyl alcohol (190 μL, 3.00 mmol) in anhydrous dichloromethane (20 mL) under argon was stirred with powdered molecular sieves (4A, 1.0 g) for 2h at room temperature. The mixture was cooled to −20 °C and treated dropwise with a solution of trimethylsilyl triflate (18 μL, 1.0 mmol) in anhydrous CH_2_Cl_2_ (5 mL) and monitored by thin-layer chromatography. After 30 min, the reaction was quenched with triethylamine (1 mL), diluted with dichloromethane (20 mL), and filtered via Celite. The filtrate was washed with HCl (1 N, 20 mL), water (20 mL), and saturated NaCl solution (20 mL). The organic phase was dried via Na_2_SO_4_, evaporated and the sirupy residue purified by column chromatography on silica gel with dichloromethane-methanol (30:1) to give **26** (560 mg, 83%) as colorless solid, *R*_f_ = 0.32 (PE/EE 1:1); mp 113 °C; [α]_D_^20^ = +45.3 (*c* = 1.0 in CHCl_3_); ^1^H-NMR (400 MHz, CDCl_3_) δ = 5.84 (ddd, *J*_2,3all_ = 5.5, *J*_cis_ = 10.7, *J*_trans_ = 16.7 Hz, 1H, H_All-2_), 5.40 (d, *J*_1’,2’_ = 3.8 Hz, 1H, H-1′), 5.35 (dd, *J =* 8.25, 8.25 Hz, 1H, H-3′), 5.30–5.16 (m, 3H, H-3, HAll-3), 5.04 (t, *J =* 9.7, 9.7 Hz, 1H, H-4′), 4.91–4.80 (m, 2H, H-2, H-2′), 4.57 (d, *J*_1,2_ = 7.7 Hz, 1H, H-1), 4.47 (dd, *J*_6ab_ = 12.1 Hz, 1H H-6a), 4.34–4.16 (m, 3H, H_All-1a_), 4.15–3.89 (m, 5H, H-5′, H_All-1b_), 3.75–3.58 (m, 1H, H-5), 2.14–2.00 (7 × s, 7 × 3H, H_Ac_); MALDI-TOF: *m*/*z calcd.* for C_29_H_40_O_18_ 676.22, found: 699.5 [*M* + Na]^+^.

#### 3.2.4. (*E*)-4-(4-Dimethoxymethylphenoxy)-but-2-enyl 2,3,6-Tri-*O*-acetyl-4-*O*-(2,3,4,6-tetra-*O*-acetyl-α-d-glucopyranosyl)-β-d-glucopyranoside (**27**)

Under Schlenk conditions, allyl glycoside **26** (412 mg, 609 μmol) and para-allyloxybenzaldehyde dimethylacetal **22** (1.27 g, 6.10 mmol) were dissolved in anhydrous and degassed dichloromethane (50 mL), and freshly activated molecular sieves 4A (500 mg) were added. Methathesis catalyst Grubbs-Hoveyda 2nd generation **23** (39 mg, 61 mmol) dissolved in dry and degassed dichloromethane (1 mL), was added by syringe to obtain a 0.02 m solution. The reaction mixture was heated under reflux for 6 h. Conversion of the starting material was monitored by TLC. The solution was concentrated under reduced pressure, and the crude product was directly purified by column chromatography using silica and a petroleum ether/ethyl acetate gradient to give **27** (396 mg, 76%) as colorless syrup; *R*_f_ = 0.30 (PE/EE 1:1); [α]_D_^20^ = +23.8 (*c* = 1.0 in CHCl_3_); ^1^H-NMR (400 MHz, CDCl_3_) δ = 7.34 (d, *J*_Ar_ = 8.5 Hz, 2H, H-1_arom_), 6.88 (d, *J*_Ar_ = 8.5 Hz, 2H, H-2_arom_), 5.95–5.80 (m, 2H, H-2_bu_, H-3_bu_), 5.40 (d, *J*_1’,2’_ = 3.8 Hz, 1H, H-1’), 5.36–5.33 (m, 2H, H-3’, H_acetal_), 5.20 (dd, *J*_2,3_ = 9.8, *J*_3,4_ = 7.8 Hz, 1H, H-3), 5.04 (dd, *J*_3’,4’_ = 9.8, *J*_4’,5’_ = 9.9 Hz, 1H, H-4’), 4.91–4.80 (m, 2H, H-2, H-2’), 4.59 (d, *J*_1,2_ = 7.9 Hz, 1H, H-1), 4.47 (dd, *J*_6ab_ = 12.1 Hz, 1H H-6a), 4.43–4.40 (m, 2H, H-1_bu_), 4.34–4.16 (m, 2H, H-4_bu_), 4.15–3.89 (m, 3H, H-6′ab, H-5’), 3.66 (ddd, *J*_4,5_ = 9.8, *J*_5,6a_ = 3.4, *J*_5,6b_ = 6.9 Hz, 1H, H-5), 3.30 (s, 6H, OCH_3_), 2.14–2.00 (7 × s, 7 × 3H, HAc); ^13^C-NMR (126 MHz, CDCl_3_) δ = 170.4, 170.3, 170.2, 169.8, 169.5, 169.2, 169.0 (7 × s 7 × 3H, CH_3_**C**O), 132.0, 129.3, 128.0, 126.9 (4 × s 4 × 1H, C_arom_), 114.9 (C-2_bu_), 114.5 (C-3_bu_), 105.5 (C_acetal_), 102.7 (C-1’), 98.3 (C-1), 76.1 (C-4), 72.9 (C-5), 72.7 (C-3’), 72.1 (C-2), 72.0 (C-5’), 71.6 (C-2’), 71.6 (C-3), 68.0 (C-1_bu_) 67.7 (C-4’) 67.5 (C-4_bu_), 61.8 (C-6), 61.5 (C-6’), 52.0 (O**C**H_3_), 20.8, 20.7, 20.6, 20.5 (4 × 1C, **C**H_3_CO); HRMS (FAB): *m/z calcd.* for C_39_H_53_O_21_^+^: 857.3074, found: 857.3100 [M + H]^+^.

#### 3.2.5. 4-(4-Formylphenoxy)-butyl 4-*O*-(α-d-Glucopyranosyl)-β-d-glucopyranoside (**12**)

The metathesis product **27** (300 mg, 350 μmol) was dissolved in a mixture of THF, water, and TFA (90:9.9:0.1 *v*/*v*) to obtain a 0.2 M solution. After stirring for 1 h, the reaction mixture was diluted with dichloromethane (20 mL), and the reaction was stopped by the addition of triethylamine (2 mL) followed by the addition of water (20 mL). The organic layer was separated and dried over Na_2_SO_4_. The solvent was removed under reduced pressure, and the crude product was used directly without further purification. For hydrogenolysis, the aldehyde was dissolved in anhydrous ethyl acetate (10 mL) in a flame-dried flask containing palladium (10%) on charcoal and diphenylsulfide (0.01 eq). The suspension was degassed, and after purging with hydrogen, the mixture was stirred for 12 h. Then the suspension was filtered and thoroughly dried before the residue was redissolved in dry THF (10 mL) and cooled to 0° C, followed by the addition of acetic acid (5 eq). After warming to room temperature, 1 M TBAF solution in THF (1.2 eq) was added and the mixture stirred overnight. After termination (TLC control), the reaction was quenched by the addition of brine and extracted with ethyl acetate (4 × 100 mL). The combined organic layers were concentrated, redissolved in methanolic sodium methoxide solution (20 mL, 0.1 M), and stirred for 6h at room temperature. The mixture was neutralized with Amberlite IR 120 (H^+^) resin, filtered, and evaporated. The crude product was purified by flash chromatography on silica with dichloromethane/methanol (3:1) to give product **12** (139 mg, 77%) as a colorless solid. *R*_f_ = 0.20 (CH_2_Cl_2_/MeOH 3:1); mp: 140 °C; [α]_D_^20^ = −9.8 (*c* = 1.0 in MeOH); ^1^H-NMR (500 MHz, CDCl_3_): δ = 9.84 (s, 1H, H_ald_), 7.88 (d, *J*_Ar_ = 8.6 Hz, 1H, H-1_arom_), 7.10 (d, *J*_Ar_ = 8.6 Hz, 1H, H-2_arom_), 4.56 (d, *J*_1,2_ = 7.9 Hz, 1H, H-1), 4.43 (d, *J*_1,2_ = 7.9 Hz, 1H, H-1’) 4.20 (t, *J_3bu,4bu_* = 6.6 Hz, 2H, H-4_bu_), 3.86–3.50 (m, 6H, H-1_bu_, H-6, H-6’), 3.50–3.30 (m, 8H, H-2, H-3, H-4, H-5, H-2’, H-3’, H-4’, H-5’), 1.90–1.85 (m, 2H, H-3_bu_), 1.84–1.76 (m, 2H, H-2_bu_); ^13^C-NMR (126 MHz, CDCl_3_): δ =192.9 (C_ald_), 129.0, 122.3, 115.1 (3C, C_arom_), 102.2 (C-1), 96.3 (C-1’), 79.7 (C-4), 77.1 (C-3’), 77.0 (C-5’) 75.0 (C-2’), 74.0 (C-5), 73.8 (C-3’), 73.5 (C-2), 71.2 (C-4’), 68.2 (C-1_bu_), 67.6 (C-4_bu_), 63.9 (C-6’), 63.0 (C-6), 27.3 (C-2_bu_), 27.0 (C-3_bu_); HRMS (FAB): *m*/*z calcd.* for C_23_H_35_O_13_^+^: 519.2072, found: 519.2075 [M + H]^+^.

#### 3.2.6. Allyl-tetraethylene-glycol (**29**)

To a solution of tetraethylene glycol (**28**, 3.41 g, 17.6 mmol) in anhydrous DMF (20 mL), sodium hydride (510 mg, 21.1 mmol) was added slowly. After stirring for 1 h at room temperature, the suspension was cooled to 0 °C, and allyl bromide (760 µL, 8.80 mmol) was added. After warming to room temperature, the mixture was stirred for 12 h. The reaction was quenched with saturated aqueous NH_4_Cl (10 mL) and diluted with ethyl acetate (20 mL). The aqueous layer was extracted with ethyl acetate (3 × 20 mL), the combined organic layers were washed with saturated NaCl solution, and dried over Na_2_SO_4_. After filtration and evaporation, the crude product was purified by column chromatography on silica gel (dichloromethane/ methanol 20:1) to yield **29** as a yellowish oil (1.86 g, 90%). *R_f_* = 0.38 (CH_2_Cl_2_/MeOH 15:1); ^1^H- NMR (400 MHz, CDCl_3_) δ = 5.90 (tdd, 1H, *J*_1a,2_ = 17.4, *J*_1b,2_ = 10.3, *J*_2,3_ = 4.9 Hz, H-2), 5.26 (d, 1H, *J*_1a,2_ = 17.4 Hz, H-1a), 5.20 (d, 1H, *J*_1b,2_ = 10.3 Hz, H-1b), 4.00 (d, 2H, *J*_2,3_ = 4.9 Hz, H-3), 3.69–3.63 (m, 2H, H-4), 3.63–3.57 (m, 10H, H-5,-6,-7,-8,-9), 3.56–3.51 (m, 4H, H-10, -11), 2.67 (s, 1H, OH); ^13^C- NMR (100 MHz, CDCl_3_) δ = 134.6 (C-2), 117.0 (C-1), 72.4 (C-3), 72.1 (C-4), 71.1 -69.9 (C-6,-7,-8,-9,-10), 69.3 (C-5), 61.7 (C-11).

#### 3.2.7. Allyl-(ω-*O*-phthalimido)-tetraethylene-glycol (**30**)

Under argon atmosphere, derivative **29** (1.10 g, 4.70 mmol), triphenylphosphine (1.85 g, 7.05 mmol), and *N*-hydroxy-phthalimide (1.15 g, 7.05 mmol) dissolved in anhydrous THF (80 mL) were treated with diisopropyl azodicarboxylate (1.43 g, 7.05 mmol) in anhydrous THF (20 mL). After stirring for 4 h, filtration, evaporation, and purification by column chromatography on silica gel (petroleum ether/ethyl acetate 1:1), **30** was obtained as a colorless oil (1.50 g, 56%). *R_f_* = 0.31 (petroleum ether/ethyl acetate 1:1); ^1^H-NMR (400 MHz, CDCl_3_) δ = 7.87–7.80 (m, 2H, Phth-ortho), 7.76–7.70 (m, 2H, Phth-meta), 5.97 (tdd, 1H, *J*_1a,2_ = 17.1, *J*_1b,2_ = 10.5, *J*_2,3_ = 5.4 Hz, H-2), 5.24 (d, 1H, *J*_1a,2_ = 17.1 Hz, H-1a), 5.17 (d, 1H, *J*_1b,2_ = 10.5 Hz, H-1b), 4.40–4.37 (m, 2H, H-11), 4.00 (d, 2H, *J*_2,3_ = 5.4 Hz, H-3), 3.89–3.84 (m, 2H, H-10), 3.68–3.57 (m, 12H, H-4,-5,-6,-7,-8,-9); ^13^C-NMR (100 MHz, CDCl_3_) δ = 163.4 (C-Carboxy), 134.7 (C-2), 133.9 (C-Phth-meta), 128.9 (C-Phth-ipso), 123.9 (C-Phth-ortho), 117.0 (C-1), 77.2 (C-11), 72.0–69.9 (C-3,4,5,6,7,8,9), 68.9 (C-10). MALDI-TOF *m/z calcd* for C_19_H_25_NO_7_: 379.16, found: 402.50 [*M* + Na]^+^.

#### 3.2.8. Allyl-(ω-hydroxylamino)-tetraethylene-glycol (**31**)

Compound **30** (1.45 g, 3.82 mmol) dissolved in anhydrous acetonitrile (30 mL) was treated with hydrazine hydrate (880 mL, 19.1 mmol). After stirring for 2h at room temperature, the white suspension was evaporated and purified by column chromatography on silica gel (dichloromethane/ methanol 10:1) to give **31** as a colorless oil (750 mg, 75%). *R_f_* = 0.31 (petroleum ether/ ethyl acetate 1:1); ^1^H-NMR (400 MHz, CDCl_3_) δ = 5.99 (tdd, 1H, *J*_1a,2_ = 10.3, *J*_1b,2_ = 17.2, *J*_2,3_ = 5.1 Hz, H-2), 5.45 (s, 2H, ONH_2_), 5.28 (d, 1H, *J*_1b,2_ = 17.2 Hz, H-1b), 5.19 (d, 1H, *J*_1a,2_ = 10.3 Hz, H-1a), 4.00 (d, 2H, *J*_2,3_ = 5.1 Hz, H-3), 3.84–3.79 (m, 2H, H-11), 3.70–3.64 (m, 2H, H-10), 3.62–3.50 (m, 12H, H-4,-5,-6,-7,-8,-9); ^13^C-NMR (100 MHz, CDCl_3_) δ = 133.9 (C-2), 117.0 (C-1), 74.2 (C-11), 72.0–68.9 (C-3,-4,-5,-6,-7,-8,-9,-10); MALDI-TOF: *m*/*z calcd* for C_11_H_23_NO_5_: 249.16, found: 272.9 [*M* + Na]^+^.

#### 3.2.9. Aminooxy-(ω-3-thioacetylpropyl)-tetraethylene-glycol (**1**)

To a stirred solution of alkene **31** (600 mg, 2.41 mmol) in anhydrous THF (10 mL), thioacetic acid (850 µL, 12.0 mmol) and a catalytic amount of AIBN were added. The mixture was irradiated for 3 h with UV light. Then the solvent was removed under reduced pressure and the yellowish crude was purified by column chromatography on silica gel (CH_2_Cl_2_/ methanol 20: 1). Compound **1** was obtained as a yellowish oil (780 mg, quant.). *R_f_* = 0.28 (CH_2_Cl_2_/ MeOH 10:1); ^1^H-NMR (400 MHz, CDCl_3_) δ = 5.44 (s, 2H, ONH_2_), 3.74–3.70 (m, 2H, H-11), 3.68–3.51 (m, 16H, H-3,-4,-5,-6,-7,-8,-9,-10), 3.08 (t, 2H, *J*_1,2_ =6.4 Hz, H-1), 2.33 (s, 3H, Sac), 2.13–1.98 (m, 2h, H-2); ^13^C-NMR (100 MHz, CDCl_3_) δ = 72.3 (C-3), 71.3–68.9 (C-4,-5,-6,-7,-8,-9,-10), 74.2 (C-11), 30.5 (C-1), 28.6 (Sac), 21.2 (C-2). MALDI-TOF *m*/*z calcd* for C_11_H_23_NO_5_: 325.16, found: 348.0 [*M* + Na]^+^.

#### 3.2.10. 3-Thioacetylpropyl-tetraethylene-glycol (**2**)

According to the synthesis of compound **1,** alkene **29** (680 mg, 2.90 mmol) was reacted with thioacetic acid (1.03 mL, 14.5 mmol) and AIBN (450 mg, 2.74 mmol) in anhydrous THF (10 mL). Compound **2** was obtained after column chromatography using silica gel (CH_2_Cl_2_/ methanol 20:1) as a yellowish oil (905 mg, quant.) *R_f_* = 0.40 (CH_2_Cl_2_/ MeOH 15:1); ^1^H-NMR (400 MHz, CDCl_3_) δ = 3.75–3.71 (m, 2H, H-11), 3.69–3.52 (m, 16H, H-3,-4,-5,-6,-7,-8,-9,-10), 3.09 (t, 2H, *J*_1,2_ = 6.4 Hz, H-1), 2.57 (s, 1H, OH), 2.33 (s, 3H, SAc), 2.14–1.97 (m, 2H, H-2). ^13^C- NMR (101 MHz, CDCl_3_) δ = 72.4 (C-3), 71.5–68.8 (C-4,-5,-6,-7,-8,-9,-10), 61.7 (C-11), 30.5 (C-1), 28.7 (SAc), 21.2 (C-2).

#### 3.2.11. *O*-Phthalimido-tetraethylene glycol (**32**)

Under an argon atmosphere, tetraethylene glycol **28** (187 mg, 0.97 mmol), triphenylphosphine (5.07 g, 7.5 mmol), and *N*-hydroxy-phthalimide (0.82 g, 5.5 mmol) dissolved in anhydrous THF (80 mL) were treated dropwise with a solution of diisopropyl azodicarboxylate (1.52 g, 7.5 mmol) in anhydrous THF (20 mL). Stirring for 4 h, filtration, evaporation, and purification by column chromatography on silica gel (ethyl acetate) gave **32** as a colorless oil (622 mg, 37%). ^1^H-NMR (300 MHz, CDCl_3_) δ = 7.88–7.80 (m, 2H, Phth-ortho), 7.78–7.71 (m, 2H, Phth-meta), 4.41–4.36 (m, 2H, CH**_2_**ON), 3.89–3.84 (m, 2H, OCH**_2_**CH_2_ON), 3.74–3.66 (m, 4H, OCH_2_CH_2_O), 3.66–3.57 (m, 8H, OCH_2_CH_2_O), 2.47 (t, 1H, *J*_CH2,OH_ = 6.2 Hz, OH); ^13^C-NMR (75 MHz, CDCl_3_) δ = 163.4 (C = O), 134.4 (C-Phth-meta), 128.9 (C-Phth-ipso), 123.5 (C-Phth-ortho), 77.2 (CH_2_ON), 72.4, 70.8, 70.6, 70.4, 70.3, (OCH_2_CH_2_OH), 69.2 (O**C**H_2_CH_2_ON), 61.7 (CH_2_OH); HRMS (ES): *m*/*z calcd* for C_16_H_22_NO_7_: 340.1396 [M + H], found: 340.1378 [M + H].

#### 3.2.12. *O*-Phthalimido-(ω-thiotrityl)-tetraethylene glycol (**33**)

Under an argon atmosphere, diisopropyl azodicarboxylate (607 mg, 3.00 mmol) and a suspension of polymer-bound triphenylphosphine (2.03 g, 3.0 mmol, 1.48 mmol/g) in anhydrous THF (25 mL) were mixed and stirred for 30 min at 0 °C. Then compound **32** (509 mg, 1.5 mmol) and triphenylmethanethiol (829 mg, 3.0 mmol) dissolved in anhydrous THF (20 mL) were added dropwise at 0 °C. Under stirring for 4 h, the mixture was gradually warmed to 20 °C, the resin filtered via Celite, the residue evaporated, and the material purified on silica gel by chromatography (diethyl ether-hexane, gradient 1:1 to 1:0). Compound **33** resulted as a colorless oil (699 mg, 78%). ^1^H-NMR (300 MHz, CDCl_3_) δ = 7.86–7.79 (m, 2H, o-ArH), 7.76–7.69 (m, 2H, m-ArH), 7.44–7.38 (m, 6H, H-trityl), 7.31–7.16 (m, 9H, H-trityl), 4.38–4.33 (m, 2H, C**H_2_**ON), 3.87–3.81 (m, 2H, OC**H_2_**CH_2_ON), 3.66–3.60 (m, 2H), 3.57–3.51 (m, 2H, OCH_2_CH_2_O), 3.51–3.45 (m, 2H, OCH_2_CH_2_O), 3.43–3.36 (m, 2H, OCH_2_CH_2_O), 3.28 (t, 2H, *J*_OCH2,CH2STr_ = 6.7 Hz, OC**H_2_**CH_2_STr), 2.41 (t, 2H, *J*_OCH2,CH2STr_ = 6.7 Hz, OCH_2_C**H_2_**STr); ^13^C-NMR (75 MHz, CDCl_3_) δ = 163.3 (C = O), 144.7 (C-trityl-ipso), 134.3 (C-Phth-meta), 129.5 (C-trityl), 129.0 (C-Phth-ipso), 127.8 (C-trityl), 126.6 (C-trityl-para), 123.4 (C-Phth-ortho), 77.1 (**C**H_2_ON), 70.7, 70.4, 70.3, 70.0, 69.5 (OCH_2_CH_2_O), 69.2 (O**C**H_2_CH_2_STr), 66.5 (C-trityl-quaternary), 31.6 (OCH_2_**C**H_2_STr); HRMS (ES): *m*/*z calcd* for C_35_H_36_NO_6_S: 620.2083, [*M* + Na] found: 620.2089 [*M* + Na].

#### 3.2.13. Aminooxy-(ω-thiotrityl)-tetraethylene-glycol (**34**)

Compound **33** (598 mg, 1.0 mmol) was dissolved in acetonitrile (20 mL) and stirred with hydrazine-hydrate (230 μL, 5.0 mmol) for 2 h at room temperature. The white suspension was evaporated, the residue suspended in dichloromethane, and filtered via Celite. Evaporation gave compound **34** as a colorless oil (454 mg, 97%). ^1^H-NMR (300 MHz, CDCl_3_) δ = 7.44–7.38 (m, 6H, H-trityl), 7.31–7.16 (m, 9H, H-trityl), 5.48 (s, 2H, ONH**_2_**), 3.84–3.79 (m, 2H, CH**_2_**ON), 3.69–3.64 (m, 2H, OCH**_2_**CH_2_ON), 3.63 (m, 4H, OCH_2_CH_2_O), 3.60–3.55 (m, 2H, OCH_2_CH_2_O), 3.48–3.43 (m, 2H, OCH_2_CH_2_O), 3.31 (t, 2H, *J*_OCH2,CH2STr_ = 6.9 Hz, OCH**_2_**CH_2_STr), 2.43 (t, 2H, *J*_OCH2,CH2STr_ = 6.9 Hz, OCH_2_C**H_2_**STr); ^13^C-NMR (75 MHz, CDCl_3_) δ = 144.8 (C-trityl-ipso), 129.6 (C-trityl), 127.8 (C-trityl), 126.6 (C-trityl-para), 74.8 (**C**H_2_ON), 70.6, 70.5, 70.4, 70.1, 69.7, 69.6 (**C**H_2_O), 66.6 (C-trityl-quaterny), 31.6 (**C**H_2_STr); HRMS (ES): *m*/*z calcd* for C_27_H_34_NO_4_S: 468.2209 [M + H], found: 468.2199.

#### 3.2.14. 4-[4-(ω-Thiotrityl)-tetraethylene-glycolyl-benzaldehyde-oxime]-butyl β-d-glucopyranoside (**35**)

The aminooxy linker **34** (150 mg, 0.32 mmol) and glycoside **3** (124 mg, 0.35 mmol) [53] dissolved in acetonitrile (5 mL) were stirred with glacial acetic acid (150 μL) for 16 h at 20 °C. Following evaporation, purification was by chromatography on silica gel (dichloromethane-methanol 20:1) to give compound **35** as a colorless syrup (260 mg, quant). ^1^H-NMR (300 MHz, CDCl_3_) δ = 8.03 (s, 1H, CH = NO), 7.49 (d, 2H, *J*_ar_ *=* 8.8 Hz, H-1ar), 7.43–7.34 (m, 6H, H-trityl), 7.32–7.13 (m, 9H, H-trityl), 6.90 (d, 2H, *J*_ar_ = 8.8 Hz, H-2ar), 4.31–4.16 (m, 3H, H-1), 4.08–3.91 (m, 3H), 3.87 (dd, 1H, *J* = 11.9, 1.80 Hz), 3.80–3.72 (m, 3H), 3.67–3.58 (m, 6H), 3.57–3.50 (m, 2H), 3.45–3.36 (m, 1H), 3.36–3.12 (m, 18H), 2.37 (2 t, 4H, *J*_OCH2,CH2STr_ = 6.7, *J*_OCH2,CH2STr_ = 6.7 Hz, OCH**_2_**CH_2_STr and OCH_2_CH**_2_**STr), 1.96–1.71 (m, 4H, H-2bu,-3bu); ^13^C-NMR (75 MHz, CDCl_3_) δ = 150.0 (CH = NO), 146.3 (C-trityl-ipso), 130.8 (C-trityl), 129.6 (C-arom), 128.9 (C-trityl), 127.8 (C-trityl), 126.1 (C-arom), 115.8 (C-arom), 104.4 (C-1), 78.2 (C-4), 78.0 (C-5), 75.2 (C-2), 74.3 (CH_2_ON), 71.7, 71.6, 71.5, 71.4, 71.2, 70.7, 70.6, 70.3 (C-1bu, -2bu, CH_2_-OEG), 68.9 (C-3), 62.8 (C-6), 32.8 (CH_2_-STr), 27.4 (C-2bu), 27.0 (C-3bu); MALDI-TOF: *m*/*z calcd* for C_44_H_55_NO_11_S: 805.35, found: 828.5 [*M* + Na]^+^.

### 3.3. Formation of Saccharide-Functionalized Gold Nanoparticles

#### 3.3.1. Synthesis and Characterization of Citrate Stabilized Gold Nanoparticles

Prior to use, all glassware was cleaned thoroughly with aqua dest, aqua regia, and finally with degassed Millipore water. A solution of gold (III) chloride trihydrate (HAuCl_4_ * 3H_2_O, 158 mg, 400 µmol) in degassed Millipore water (400 mL) was heated to reflux. To this pale yellow solution, a warm (~50–60 °C) solution of sodium citrate dihydrate (447 mg, 1.52 mmol) in degassed Millipore water (40 mL) was added under reflux and vigorous stirring. The solution was stained deep red and stirred under reflux for 30 min. Then the solution was cooled to room temperature and filtered through a syringe filter unit (0.2 µm). The average diameter of the gold nanoparticles was determined by size measurements in TEM images (Ø 13.3 ± 1.3 nm) and UV-Vis spectroscopy (13.0 ± 0.4 nm). The concentration of the gold nanoparticle solution was 10.9 nM.

#### 3.3.2. Self-Assembly of Tetraethylene Glycol Linkers **1** and **2** on Gold Nanoparticles (AuNP)

The linker solution (0.06 mmol: 1. **1** 100% = 19.5 mg; 2. **1**/**2** 1:1 = 9.8 mg + 9.3 mg; 3. **1**/**2** 1:2 = 6.5 mg + 12.4 mg) in methanol (5 mL) was added to a vigorously stirred 11 nM solution of ~13 nm citrate-stabilized gold nanoparticles (120 mL, 1.32 nmol). After the addition, a color change from red to purple was visible. The mixture was stirred for 16 h at room temperature. Subsequently, the coated **Au-NPs** were purified via centrifuge filtration (Millipore Amicon Ultra, Darmstadt, Germany, 50 kDa cutoff) in 30 mL portions by dissolving in Millipore water (5 mL) and filtration (five times). After the final filtration, the nanoparticles were diluted with Millipore water (330 µL:200 µL filtration residue + 130 µL addition) to obtain 1.0 µM solutions of SAM-coated gold nanoparticles **Au-NP 1**, **Au-NP 2, Au-NP 3,** and **Au-NP 4** (total volume each 1.32 mL). These solutions could be stored at 4 °C for at least four weeks.

#### 3.3.3. Oxime Coupling of Benzaldehyde Functionalized Glycosides **3**–**20** to Aminooxy Coated Gold Nanoparticles

To a 1 M stock solution of glycosides **3**–**11**, **13–15**, and **17–20** (100 μL, 100 μmol) for **12** and **16** (50 µL, 50 µmol) in 10 mM acetate buffer (pH 4.7), a 1.0 μM solution of aminooxy-coated AuNPs (400 μL, 0.400 nmol) in Millipore water was added. These transparent deep red reaction mixtures were shaken at 40 °C for 16 h. Then the glyconanoparticles **GNP-1 –GNP 19** were purified by ten times repeating centrifuge filtration (Millipore Amicon Ultra, 50 kDa cutoff) and dilution with Millipore water. After the final filtration, the GNPs were diluted with Millipore water (400 µL: 200 µL filtration residue + 200 µL addition) to obtain 1.0 μM solutions (400 µL) of glyconanoparticles **GNP-1–GNP-19**. These solutions were storable at −20 °C for at least four weeks (storage in sterile filtrated, NaN_3_ containing Millipore water to avoid bacterial contamination).

#### 3.3.4. Determination of Carbohydrate Concentration on Gold Nanoparticles (Anthrone Test)

The amount of sugar on the GNPs can be easily determined by an anthrone-sulfuric acid assay. For this purpose, standard curves were recorded with the glycoconjugates **4** (for monosaccharides, MZ = 356.37 g/mol), **10** (for the disaccharides, MZ = 518.51 g/mol), and **14** (for trisaccharides, MZ = 705.70 g/mol). The stock solutions were diluted 1:100 (**4**: 3.56 µg/µL; **10**: 5.18 µg/µL; **14**: 7.05 µg/µL). Aliquots were taken from the dilutions to obtain a content of 7–176 µg per mL solution (see Table S1 in Supplementary Materials). The color reaction was carried out by adding 2.5 mL of a freshly prepared solution of 0.5% anthrone (wt%) in 95% sulfuric acid to 1 mL of carbohydrate solution in a test tube. The reaction mixture was carefully heated for 10 min in a boiling water bath. After cooling, the absorbance was immediately measured in the UV-Vis spectrometer at 620 nm (double determination). A blank sample was prepared without carbohydrate. To determine the content of carbohydrates on the nanoparticles, 1 µM GNP solution (40 µL/538 µg) was diluted to 1 mL and treated with anthrone-sulfuric acid as described for the preparation of the standard curves. The absorbance was measured at 620 nm. The formulas in Table S1 can be used to calculate the carbohydrate contents. The number of ligands per nanoparticle is given by the corresponding equation. The results are summarized in Table S2 in the Supplementary Materials.

#### 3.3.5. ConA Binding Study Using TEM

Transmission electron microscopy was performed on a JEOL JEM 2100F (Tokyo, Japan) equipped with a 2 k × 2 k CCD camera and an EDXS system at 200 kV. For the measurements, a drop of the respective nanoparticle solutions was applied to a carbon-coated copper carrier and dried in air. For ConA binding studies, the nanoparticle solution was incubated with ConA solution (50 µM containing 10 mM Ca^2+^ and Mn^2+^ ions) for 12 h. Additionally, a drop of uranyl acetate solution was applied. The carrier was previously hydrophilized in an oxygen plasma (discharge grid) so that the drop of the aqueous solutions was distributed homogeneously over the sample carrier. The composition of the particles was determined using energy dispersive X-ray spectroscopy (EDXS). The particle sizes were determined, and the images of the nanoparticles were processed using DigitalMicrograph software (version 1.83) from Gatan Inc. (Pleasanton, CA, USA).

## 4. Conclusions

For studies of carbohydrate-protein and carbohydrate-carbohydrate interactions (CCI/CPI), powerful tools are required. Here, we present a novel modular approach. Facile and rapid syntheses for linking spacers and carbohydrate derivatives could be developed, and enhanced binding events were realized by controlling the amount and orientation of the ligand. For immobilization on biorepulsive aminoxy functionalized gold nanoparticles by oxime formation, aldehyde-functionalized glycan structures of mono-, di-, and complex trisaccharides were synthesized, employing several facile steps including olefin metathesis. Uniform gold nanoparticles were synthesized and subsequently functionalized. Glyconanoparticles were well characterized by TEM and the anthrone method. Effective immobilization and binding studies are exemplary presented for the lectin concanavalin A. This novel method showed advantageous perspectives to be employed in various biomimetic studies of carbohydrates and carbohydrate-based array development for diagnostics and screening.

## Figures and Tables

**Table 1:** Synthesis of aminooxy-terminated core-shell gold nanoparticles ^[a]^.

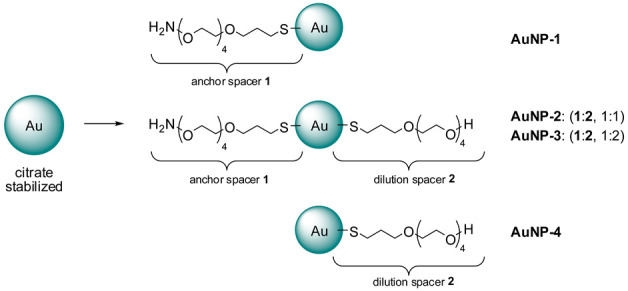			
Entry	Ratio 1:2	Product	Δλ_SP,max_ ^[b]^
1	1:0	**AuNP-1**	~4 nm
2	1:1	**AuNP-2**	~3 nm
3	1:2	**AuNP-3**	~3 nm
4	0:1	**AuNP-4**	~6 nm

^[a]^ Au-NP synthesis: HAuCl_4_, sodium citrate, H_2_O, 60 °C, 2 h; SAM formation by incubation of aqueous Au-NP solution with methanolic solutions of thioacetates **1** and **2** in different ratios for 16 h. ^[b]^ Changes in absorption maxima of surface plasmon band λ_SP,max_ occur by SAM formation on gold nanoparticles.

**Table 2:** Synthesis of aminooxy-terminated core-shell gold-glyconanoparticles ^[a]^.

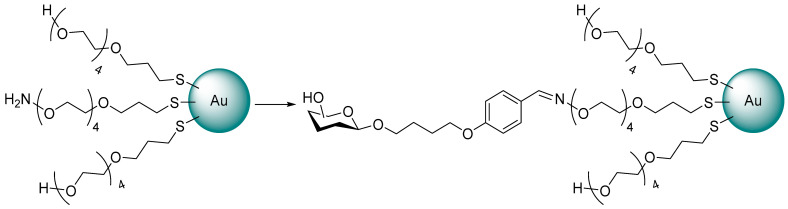					
Entry	Nanoparticle		Carbohydrate Head Group		Ligand Density ^[e]^
1	**GNP-1**	**AuNP-1** ^[b]^	β-Glucopyranose	**3**	~1280 ^[f]^
2	**GNP-2**	**AuNP-1** ^[b]^	α-Glucopyranose	**4**	~1160 ^[f]^
3	**GNP-3**	**AuNP-1** ^[b]^	α-Mannopyranose	**5**	~1260 ^[f]^
4	**GNP-4**	**AuNP-1** ^[b]^	β-Galactopyranose	**6**	~1280 ^[f]^
5	**GNP-5**	**AuNP-1** ^[b]^	α-Idopyranose	**8**	~1200 ^[f]^
6	**GNP-6**	**AuNP-1** ^[b]^	α-Talopyranose	**9**	~1220 ^[f]^
7	**GNP-7**	**AuNP-1** ^[b]^	β-Cellobiose	**10**	~1040 ^[g]^
8	**GNP-8**	**AuNP-1** ^[b]^	β-Lactose	**11**	~1060 ^[g]^
9	**GNP-9**	**AuNP-1** ^[b]^	β-Maltose	**12**	~1040 ^[g]^
10	**GNP-10**	**AuNP-1** ^[b]^	β-*N*-Acetyllactosamine	**13**	~980 ^[g]^
11	**GNP-11**	**AuNP-1** ^[b]^	Le^x^	**14**	~880 ^[h]^
12	**GNP-12**	**AuNP-2** ^[c]^	Le^x^	**14**	~660 ^[h]^
13	**GNP-13**	**AuNP-3** ^[d]^	Le^x^	**14**	~400 ^[h]^
14	**GNP-14**	**AuNP-1** ^[b]^	Le^x-Glc^	**15**	~900 ^[h]^
15	**GNP-15**	**AuNP-1** ^[b]^	Le^x-l-Gal^	**16**	~960 ^[h]^
16	**GNP-16**	**AuNP-1** ^[b]^	Le^x-β-Fuc^	**17**	~880 ^[h]^
17	**GNP-17**	**AuNP-1** ^[b]^	Le^a^	**18**	~880 ^[h]^
18	**GNP-18**	**AuNP-1** ^[b]^	Le^x-L-Rha^	**19**	~920 ^[h]^
19	**GNP-19**	**AuNP-1** ^[b]^	Le^x-d-l^	**20**	~900 ^[h]^

^[a]^ Oxime coupling by incubation of glycans in acetate buffer (pH 4.7) with Au-NP solutions for 16 h at 40 °C; ^[b]^ theoretical surface coverage of 100% glycan; ^[c]^ theoretical surface coverage of 50% glycan; ^[d]^ theoretical surface coverage of 33% glycan; ^[e]^ determination by anthrone method; ^[f]^ reference: compound **3**; ^[g]^ reference: compound **10**; ^[h]^ reference: compound **14**.

## Data Availability

The original contributions presented in this study are included in the article/Supplementary Material. Further inquiries can be directed to the corresponding authors.

## Supplementary Materials

The following supporting information can be downloaded at: https://www.mdpi.com/article/10.3390/molecules30183765/s1, Table S1. Data for determination of standard curves and formula for calculation of carbohydrate content. Figure S1. Standard curves for calculation of carbohydrate content. Table S2. Absorption after Treatment of GNPs with Anthrone Method and Calculated Ligands pro GNP.
