# Supplementary material for: Synthesis and Characterization of Glyco-SAMs on Gold Nanoparticles: A Modular Approach Towards Glycan-Based Recognition Studies
Source: Molecules. 2025 Sep 16;30(18):3765. doi: 10.3390/molecules30183765 (PMC12472522; doi:10.3390/molecules30183765)

Supporting Information

Synthesis and Characterization of Glyco-SAMs on Gold Nanoparticles: A Modular Approach towards Glycan Based Recognition Studies

Sebastian Kopitzki[1] and Joachim Thiem*[2]

Table of contents

A) Determination of the average number of carbohydrate ligands per GNP.

B) **Table S1.** Data for determination of standard curves and formula for calculation of carbohydrate content.

C) **Figure S1.** Standard curves for calculation of carbohydrate content.

D) **Table S2.** Absorption after Treatment of GNPs with Anthrone Method and Calculated Ligands pro GNP.

E) ^{1}H- and ^{13}C-NMR-Spectra of compounds **1**, **2**, **4**, **12**, **24**, **26**, **27**, **29**-**35**

A) Determination of the average number of carbohydrate ligands per GNP

The average number of carbohydrate ligands N_{CL} results from the amount of sugar n_Z per amount of glyconanoparticles n_{GNP}:

$$\boldsymbol{N_{CL} = \frac{n_Z}{n_{GNP}} = \frac{m_z\ /M_Z}{m_{GNP}\ /M_{GNP}}}$$

M_Z and m_{GNP} are given and M_{GNP} can be calculated as follows. The following simplifications are assumed for this calculation:

The molecular weight of GNPs can be considered constant and does not change significantly due to SAM occupancy and carbohydrate attachment.

For calculation of the number of gold atoms per nanoparticle, the spherical geometry of the Au-NP is assumed.

This results in the number N_{13nm} of gold atoms in nanoparticles with a diameter of 13 nm:

$$N_{13nm} = \frac{m * N_A}{M} \qquad \text{with } m = V * \rho \quad \text{and} \quad V = \frac{3}{4}\pi * r^3 = \frac{1}{6}\pi * D^3$$

$$N_{13nm} = \frac{\pi\rho * D^3}{6M} * N_A$$

Where ρ is the density of face-centered cubic gold (19.3 g/cm^3), D is the diameter of the nanoparticles (13 nm = 13 × 10^{-7} cm), M is the molecular weight of gold (196.97 g/mol) and N_A is the Avogadro number (6.023*10^{23} atoms/mol).

$$N_{13nm} = \frac{\pi * 19.3 * (13 * 10^{-7})^3}{6 * 196.97} * 6.023 * 10^{23} * \frac{g * mol * cm^3 * atoms}{cm^3 * g * mol}$$

$$N_{13nm} = 0.309 * (13 * 10^{-7})^3 * 10^{23} * atoms$$

$$N_{13nm} = 0.309 * 100 * 13^3 * atoms = \underline{\text{67.800 atoms}}$$

This results in an average rounded number of 67.800 atoms per nanoparticle.

With the number of gold atoms per nanoparticle, the molecular weight can now be calculated as:

M = atom mass (Gold) * atom number * $1.66*10^{27}$ kg

M = 196.97 u * atom number * $1.66*10^{-27}$ g/u * $6.023*10^{23}$ atoms/mol

M = 196.97 u * 67800 atoms * $1.66*10^{-27}$ g/u * $6.023*10^{23}$ atoms/mol

M = 13 458 282 g/mol ≈ <u>13.458.000 g/mol</u>

Table S1. Data for determination of standard curves and formula for calculation of carbohydrate content.

Stock solution (1:100) → µg compound **3** / mL	$A_{620\ nm}$	Stock solution (1:100) → µg compound **10** / mL	$A_{620\ nm}$
25 µL → 89 µg	0.453	25 µL → 129.5 µg	0.756
20 µL → 71.2 µg	0.352	20 µL → 103.6 µg	0.607
15 µL → 53.4 µg	0.270	15 µL → 77.7 µg	0.448
10 µL → 35.6 µg	0.181	10 µL → 51.8 µg	0.301
5 µL → 17.8 µg	0.089	5 µL → 25.9 µg	0.155
2 µL → 7.1 µg	0.039	2 µL → 10.4 µg	0.060
**Stock solution (1:100) → µg compound 14 / mL**	**$A_{620\ nm}$**		
25 µL → 176.3 µg	1.095	$content_{Mono}$ [µg] = $A_{620\ nm}$ / 0.0062	
20 µL → 141 µg	0.873		
15 µL → 105.8 µg	0.654	$content_{Di}$ [µg] = $A_{620\ nm}$ / 0.0058	
10 µL → 70.5 µg	0.441		
5 µL → 35.3 µg	0.215	$content_{Tri}$ [µg] = $A_{620\ nm}$ / 0.0050	
2 µL → 14.1 µg	0.092		

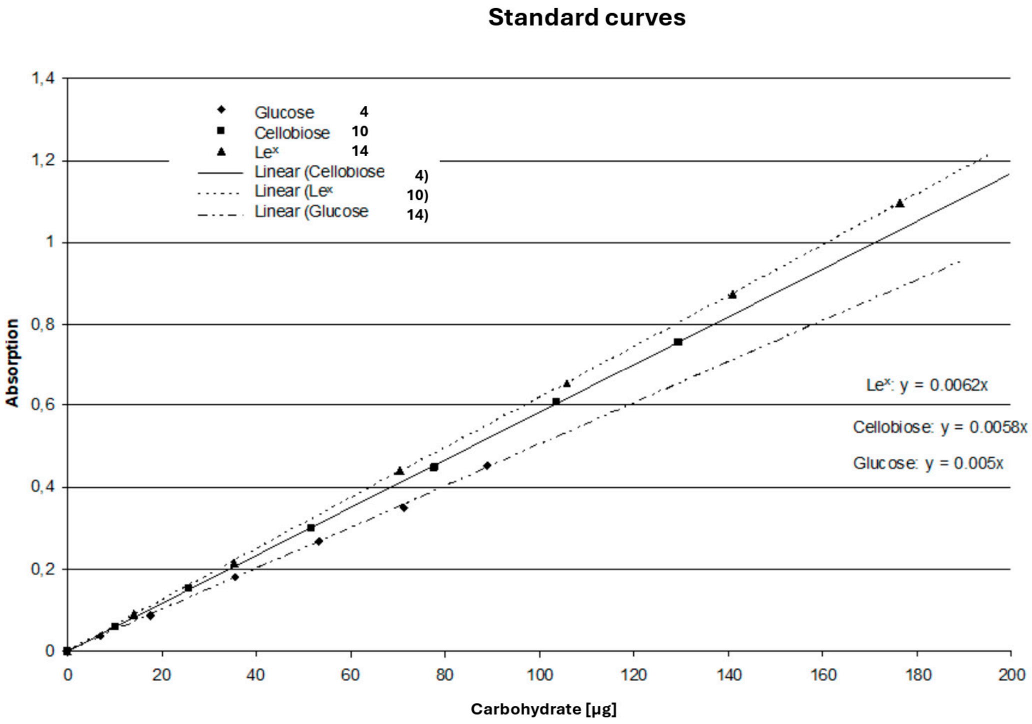

Figure S1. Standard curves for calculation of carbohydrate content.

Table S2. Absorption after Treatment of GNPs with Anthrone Method and Calculated Ligands pro GNP.

GNP	$A_{620\ nm}$	Cont. [µg]	Lig.	GNP	$A_{620\ nm}$	Cont. [µg]	Lig.
**GNP-1[a]**	0.090	18.1	1270	**GNP-11[c]**	0.153	24.7	876
**GNP-2[a]**	0.083	16.6	1165	**GNP-12[c]**	0.117	18.9	669
**GNP-3[a]**	0.090	18.0	1260	**GNP-13[c]**	0.072	11.6	411
**GNP-4[a]**	0.091	18.2	1277	**GNP-14[c]**	0.158	25.5	904
**GNP-5[a]**	0.085	17.0	1193	**GNP-15[c]**	0.168	27.1	961
**GNP-6[a]**	0.087	17.4	1221	**GNP-16[c]**	0.155	25.0	886
**GNP-7[b]**	0.126	21.7	1046	**GNP-17[c]**	0.160	25.8	915
**GNP-8[b]**	0.127	21.9	1056	**GNP-18[c]**	0.155	25.0	886
**GNP-9[b]**	0.126	21.7	1046	**GNP-19[c]**	0.159	25.6	907
**GNP-10[b]**	0.119	20.5	989				

[a] Monosaccharide; [b] Disaccharide; [c] Trisaccharide.

E) ^{1}H- and ^{13}C-NMR-Spectra

1) Hydroxylamino-(ω-3-thioacetylpropyl)-tetraethylene-glycol (**1**)

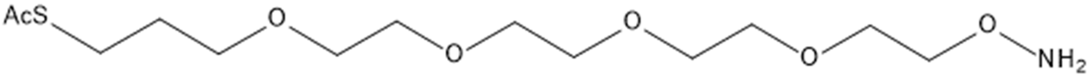

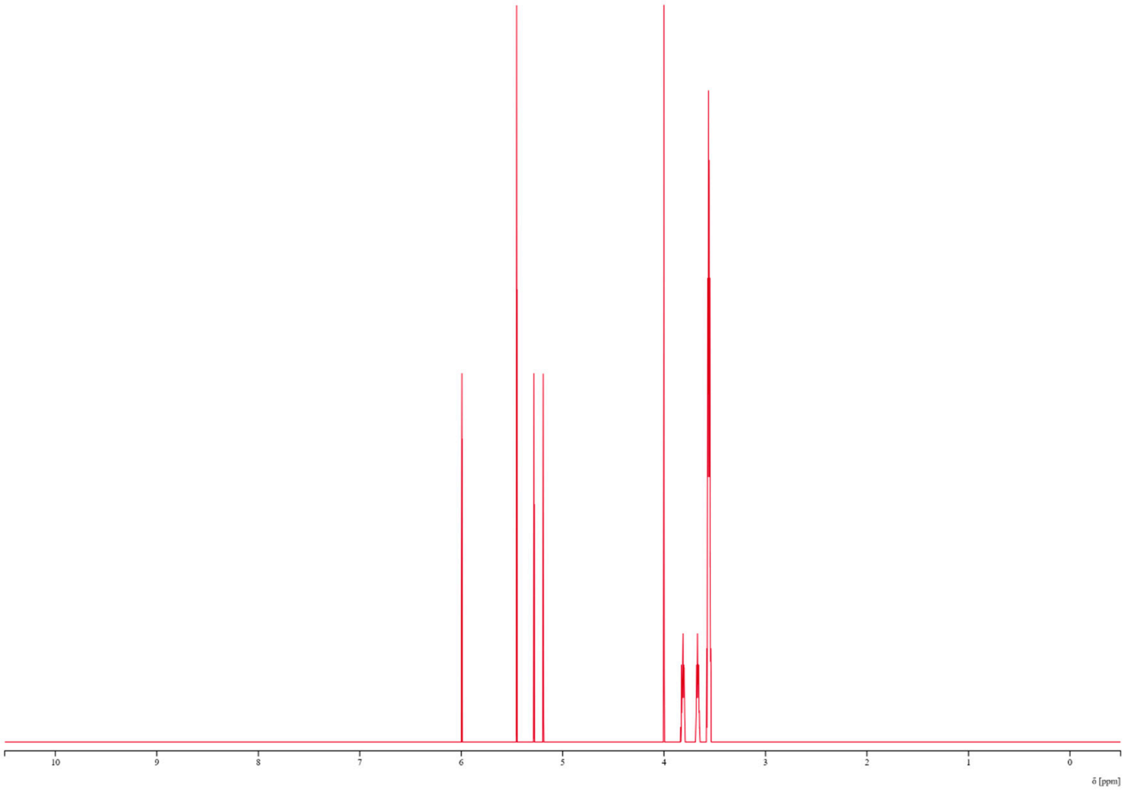

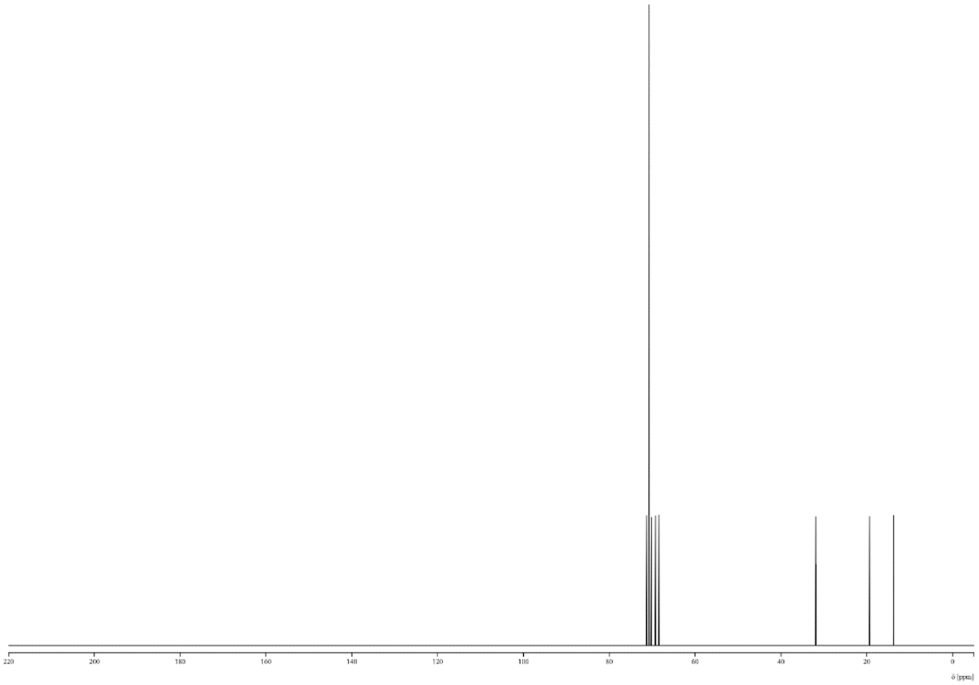

2) ω-3-Thioacetylpropyl-tetraethylene-glycol (**2**)

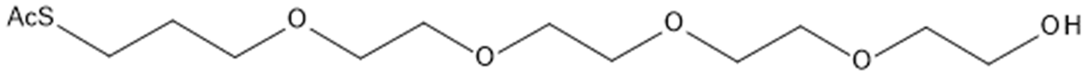

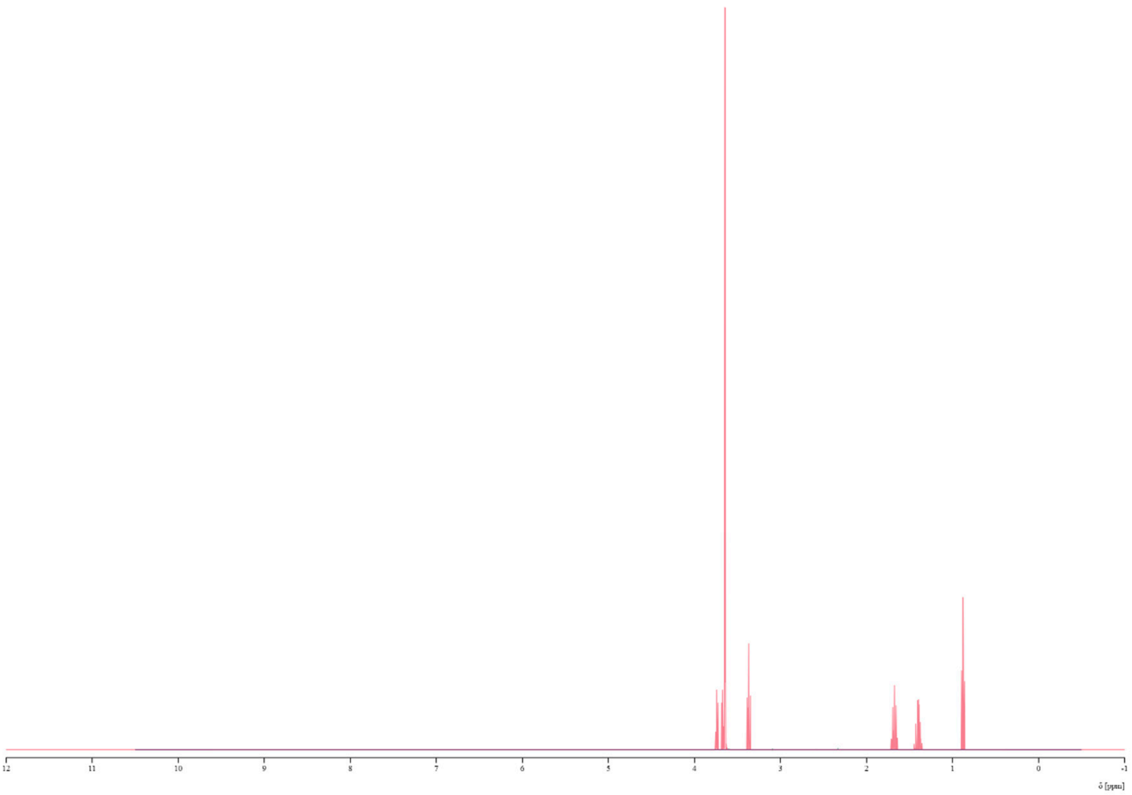

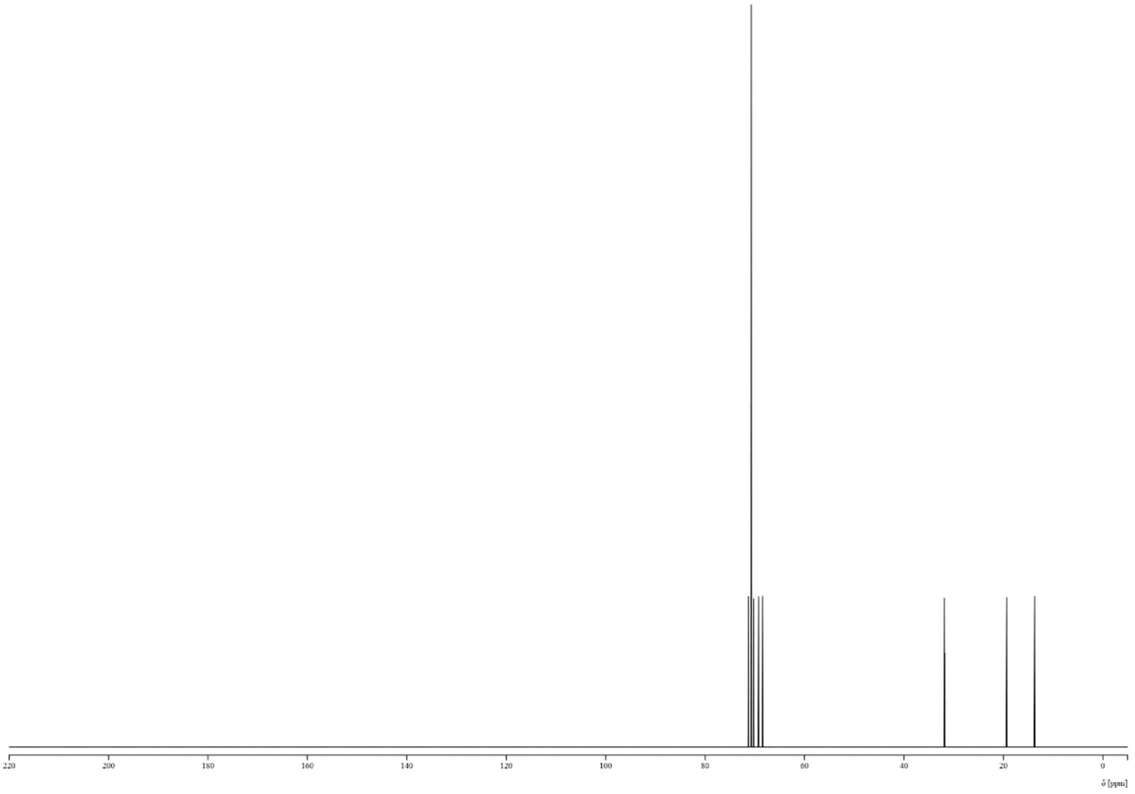

3) 4-(4-Formylphenoxy)butyl α-D-glucopyranoside (**4**)

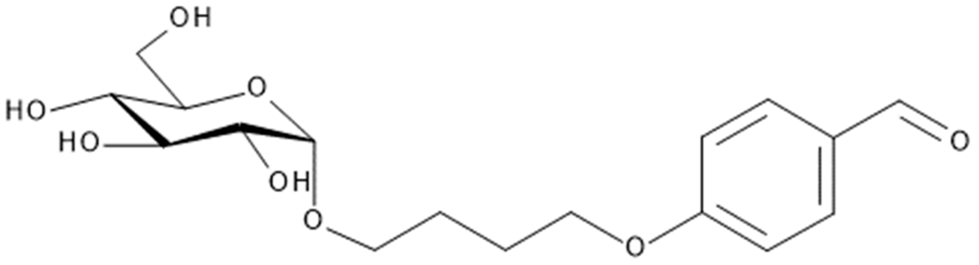

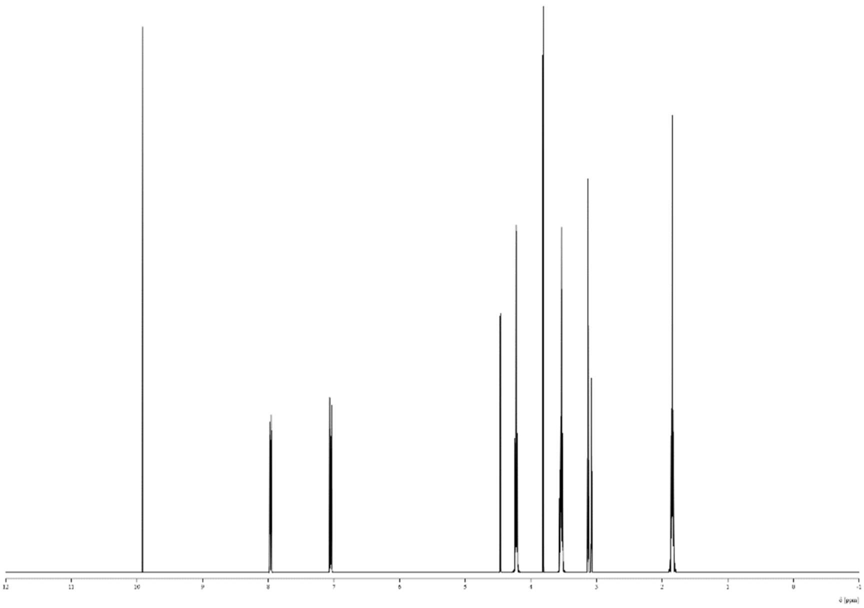

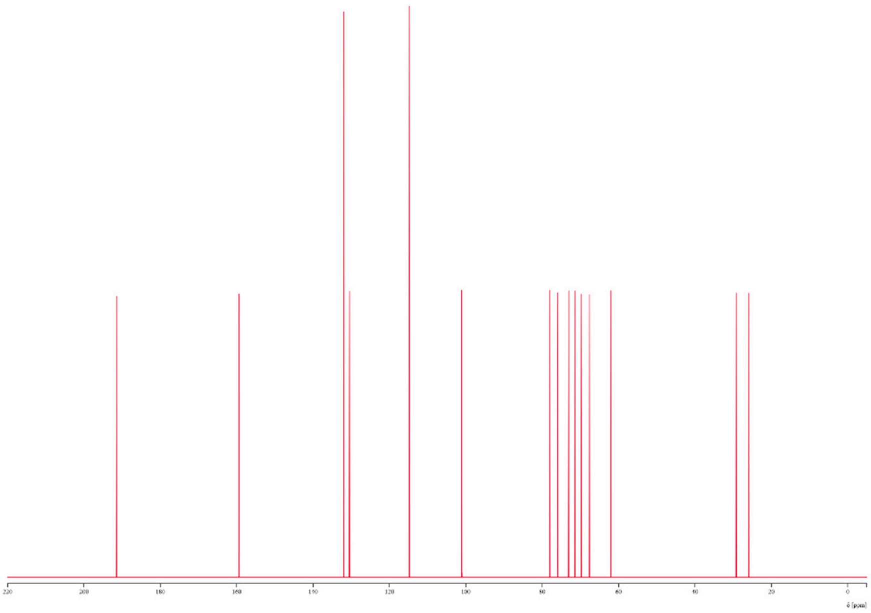

4) 4-(4-Formylphenoxy) butyl 4-*O*-(α-D-glucopyranosyl)-β-D-glucopyranoside (**12**)

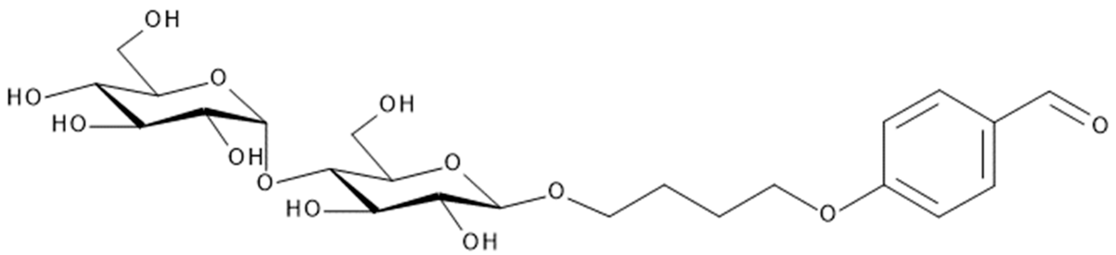

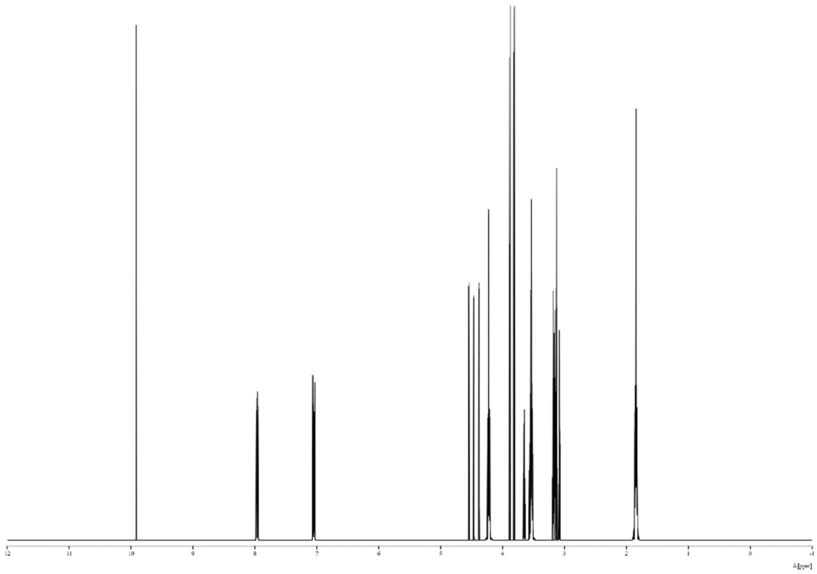

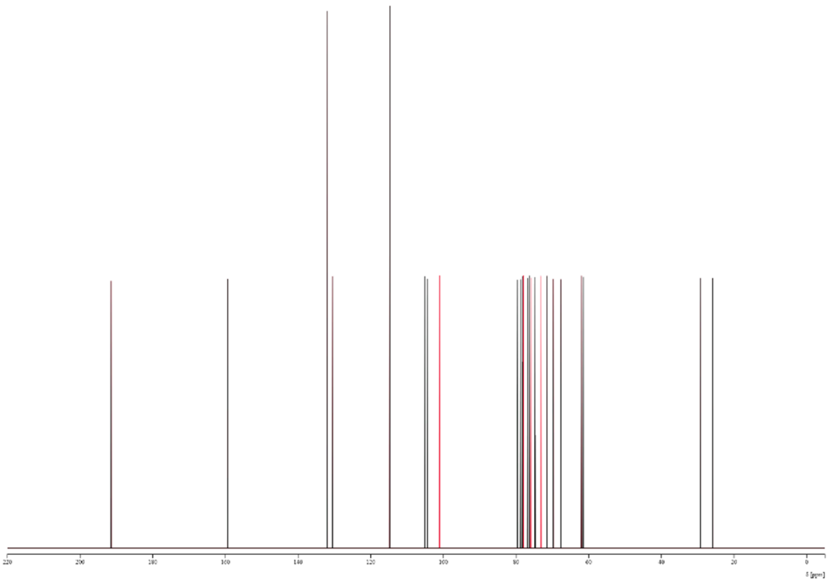

5) (*E*)-4-[4-(Dimethoxymethyl) phenoxy] but-2-enyl 2,3,4,6-tetra-*O*-acetyl-α-D-glucopyranoside (**24**)

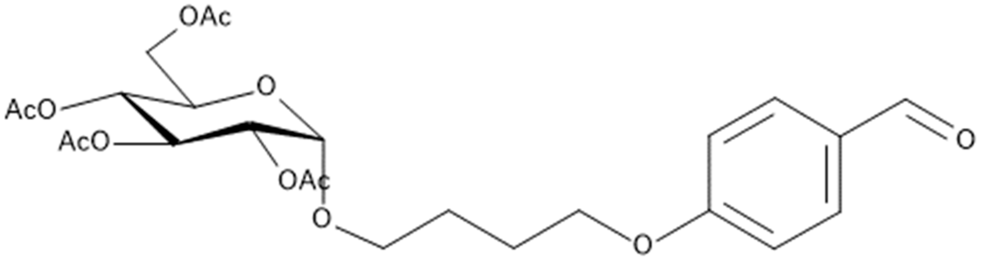

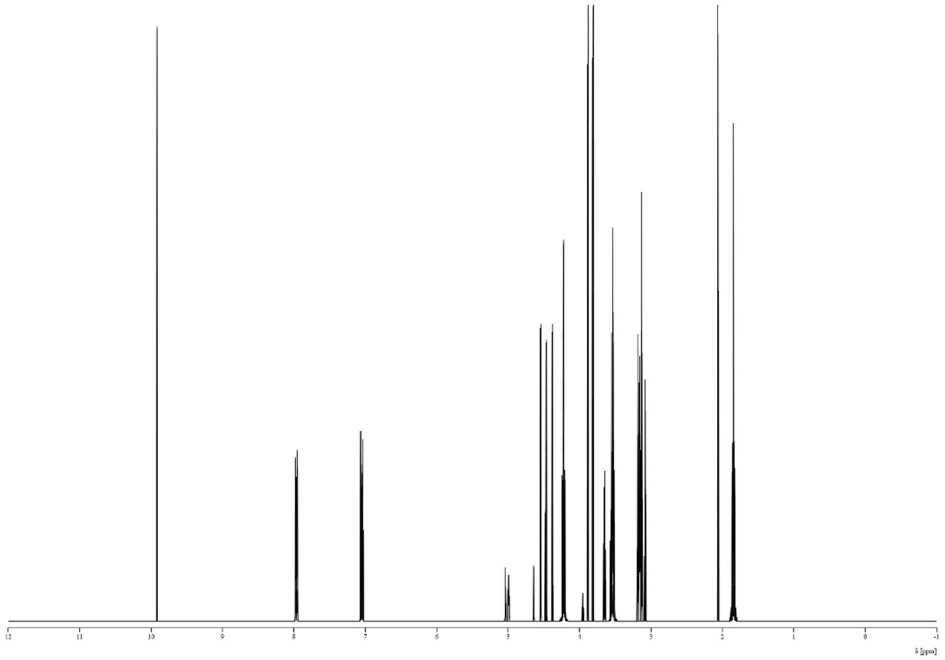

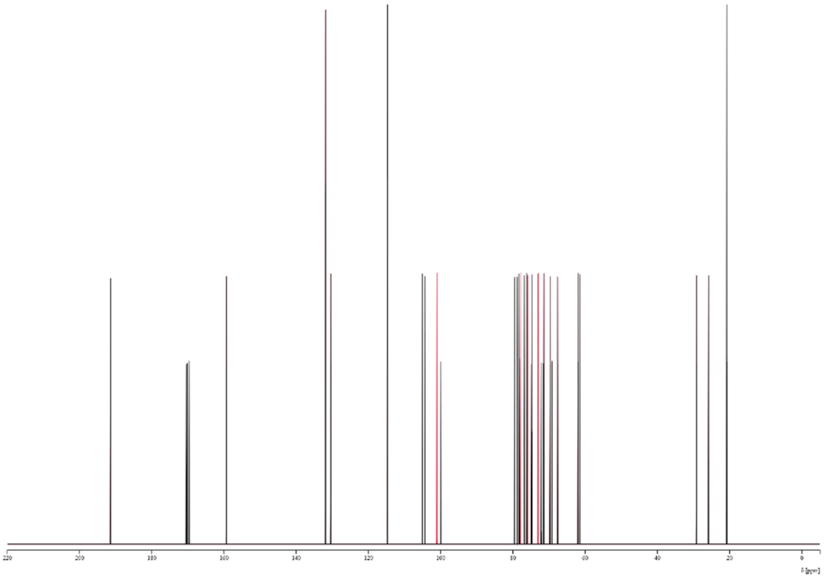

6) Allyl 2,3,6-tri-*O*-acetyl-4-*O*-(2,3,4,6-tetra-*O*-acetyl-α-D-glucopyranosyl)-β-D-glucopyranoside (**26**)

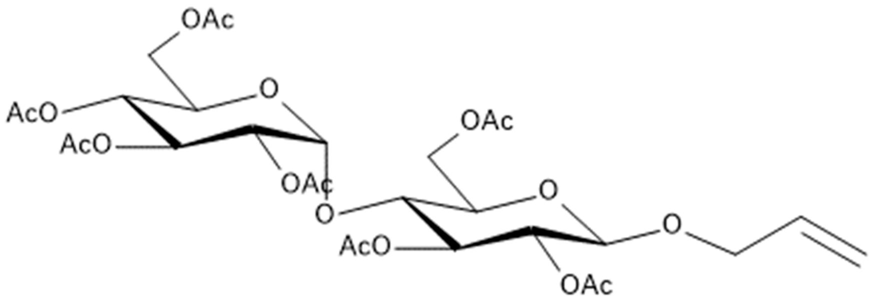

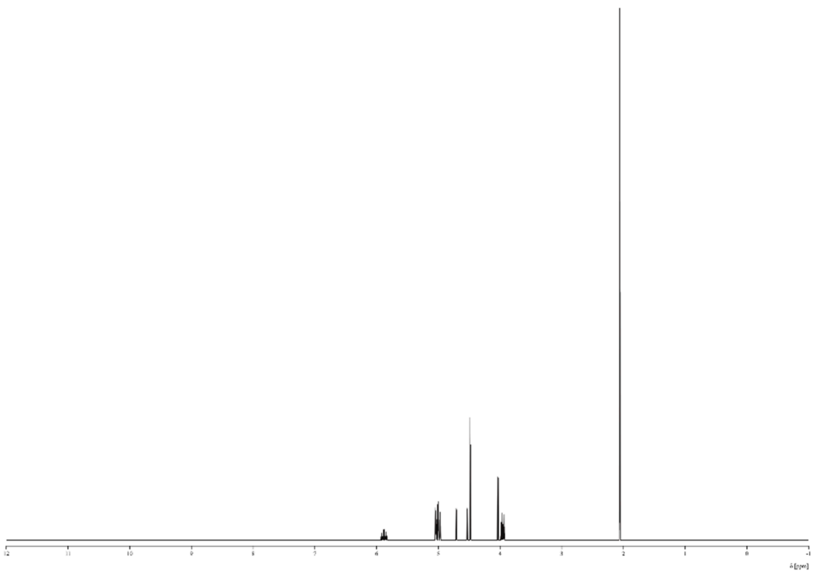

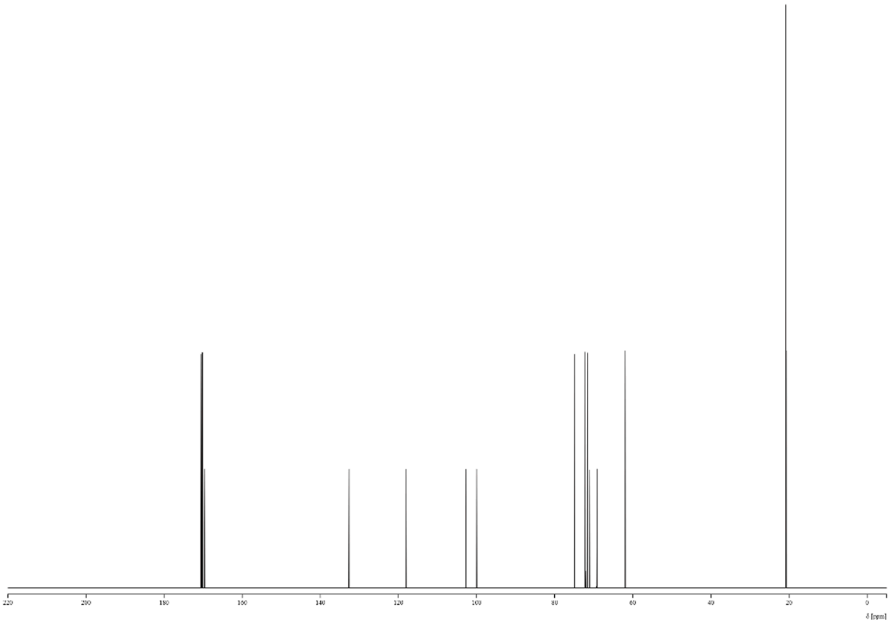

7) (*E*)-4-(4-Dimethoxymethylphenoxy)-but-2-enyl 2,3,6-tri-*O*-acetyl-4-*O*-(2,3,4,6-tetra-*O*-acetyl-α-D-glucopyranosyl)-β-D-glucopyranoside (**27**)

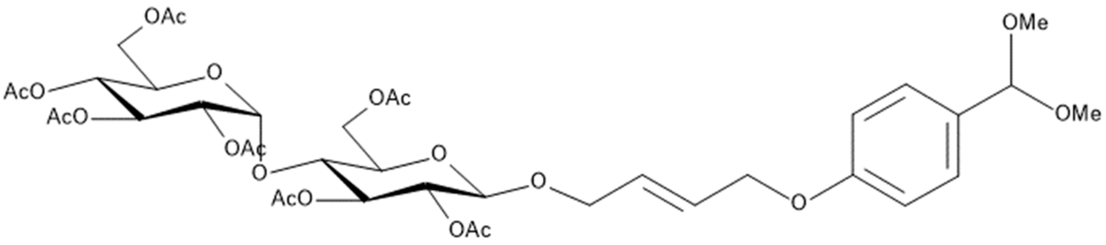

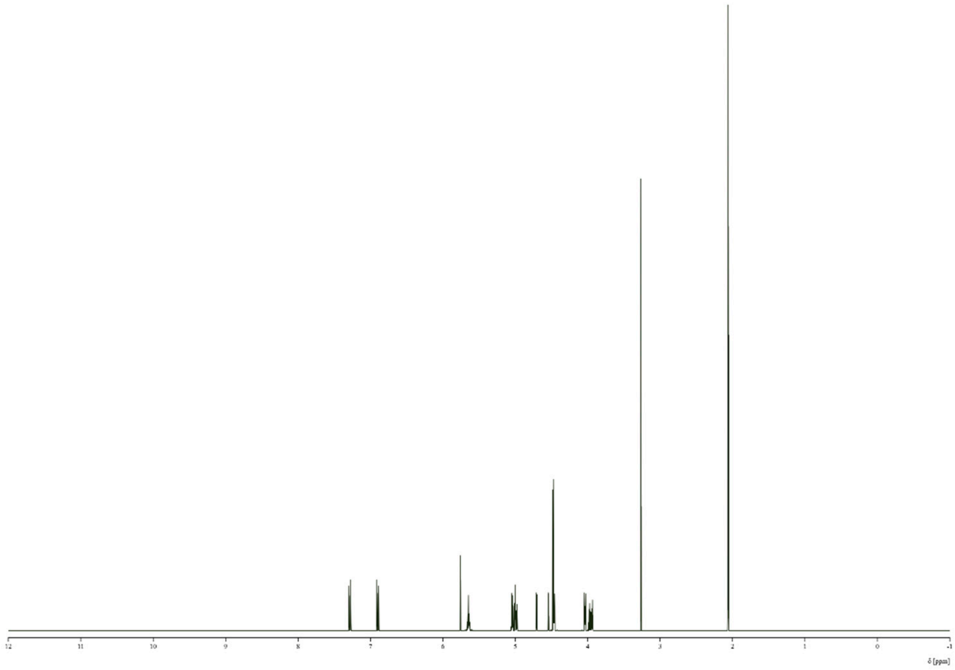

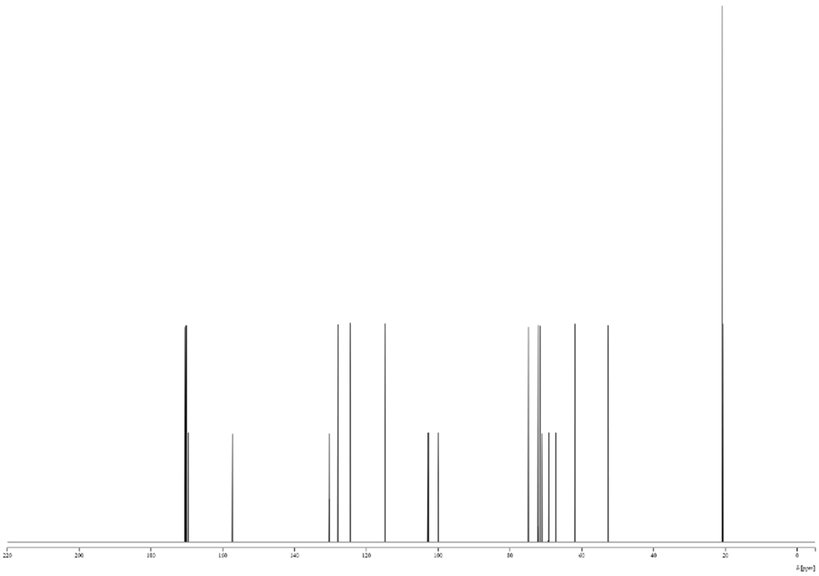

8) Allyl-tetraethylene-glycol (**29**)

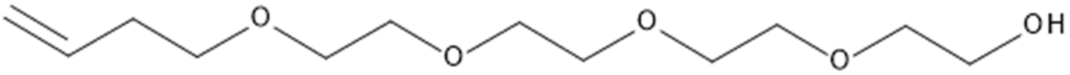

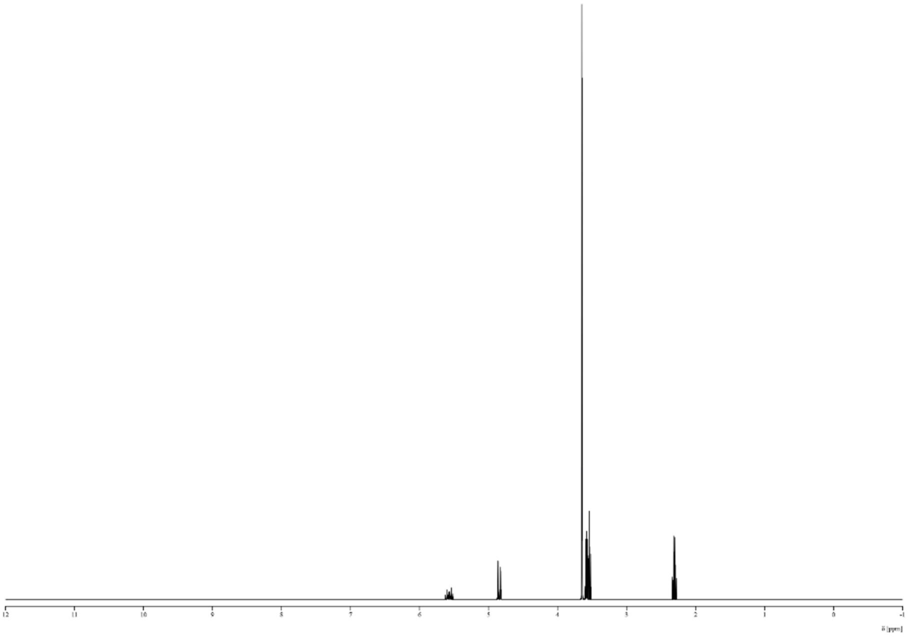

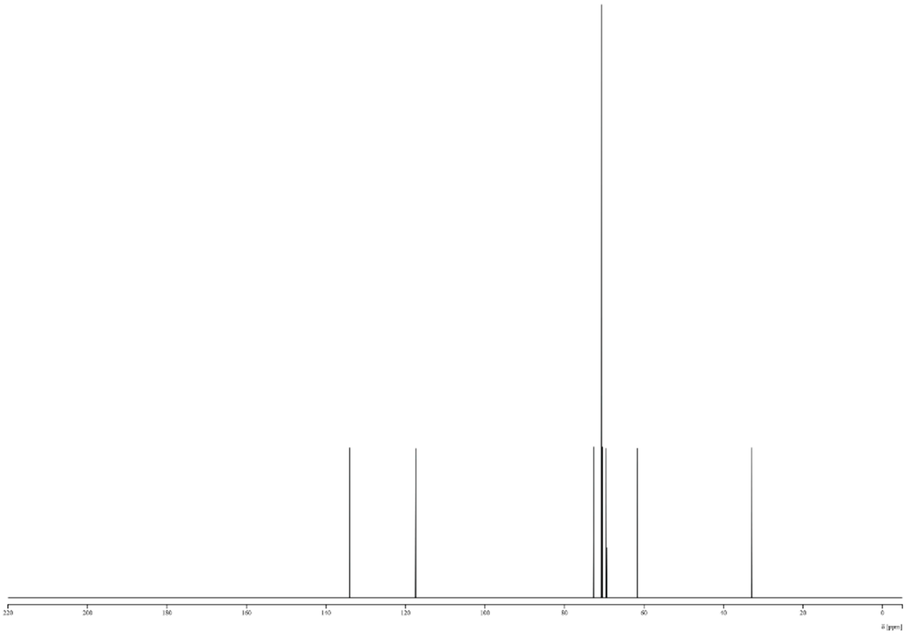

9) Allyl-(ω-*O*-phthalimido)-tetraethylene-glycol (**30**)

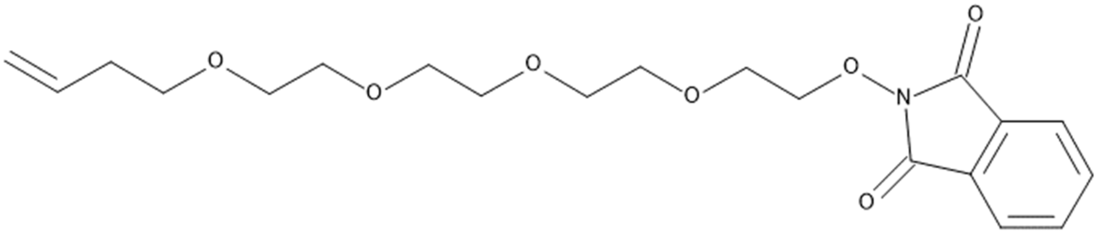

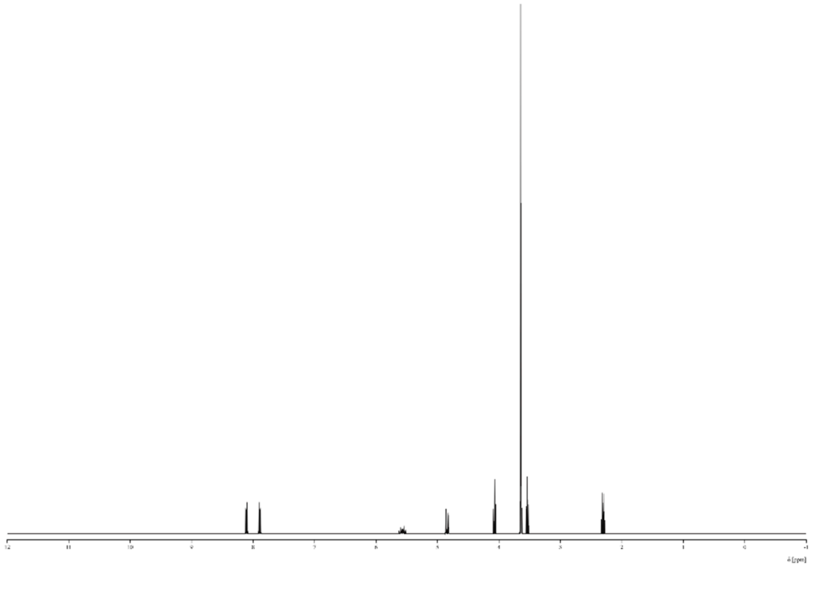

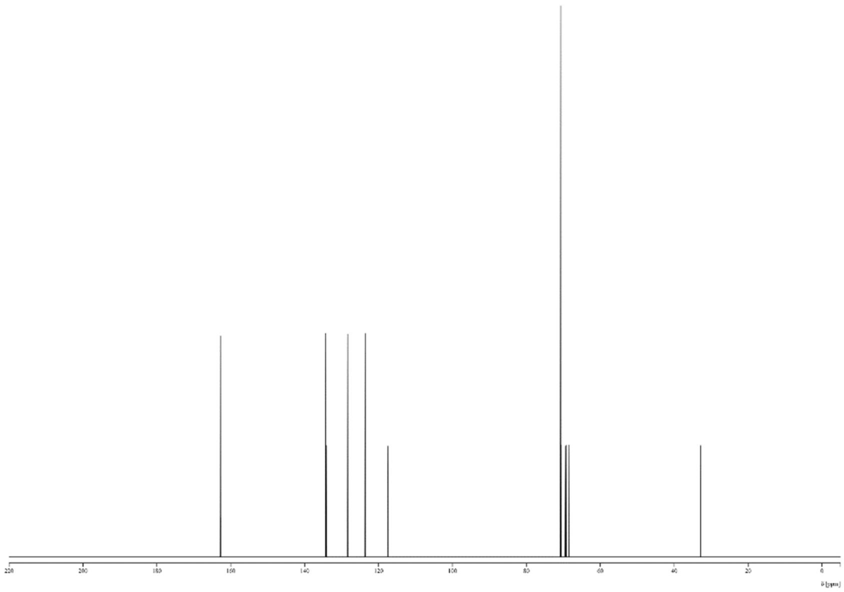

10) Allyl-(ω-hydroxylamino)-tetraethylene-glycol (**31**)

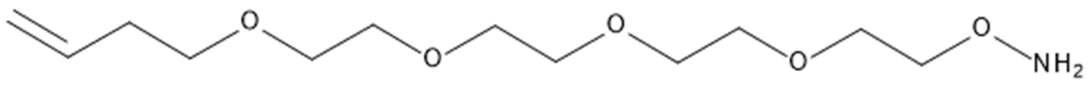

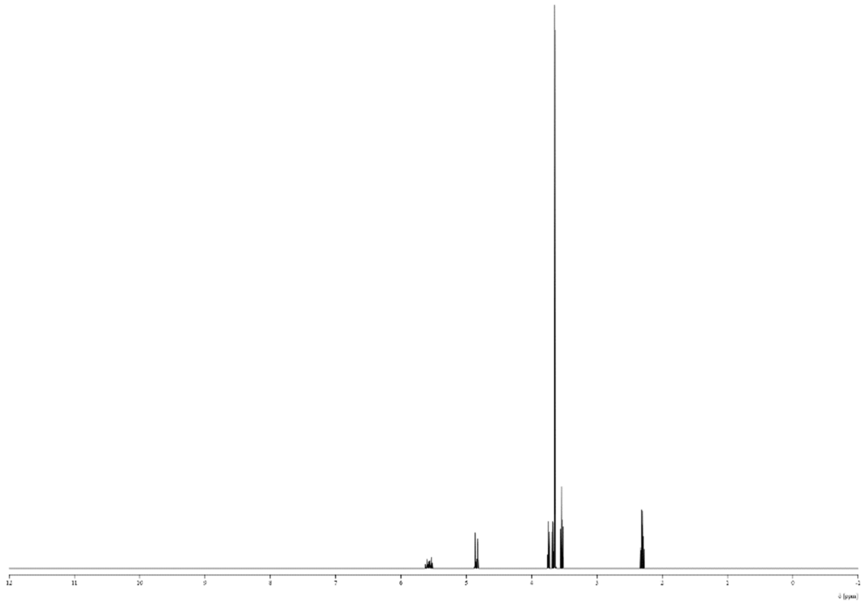

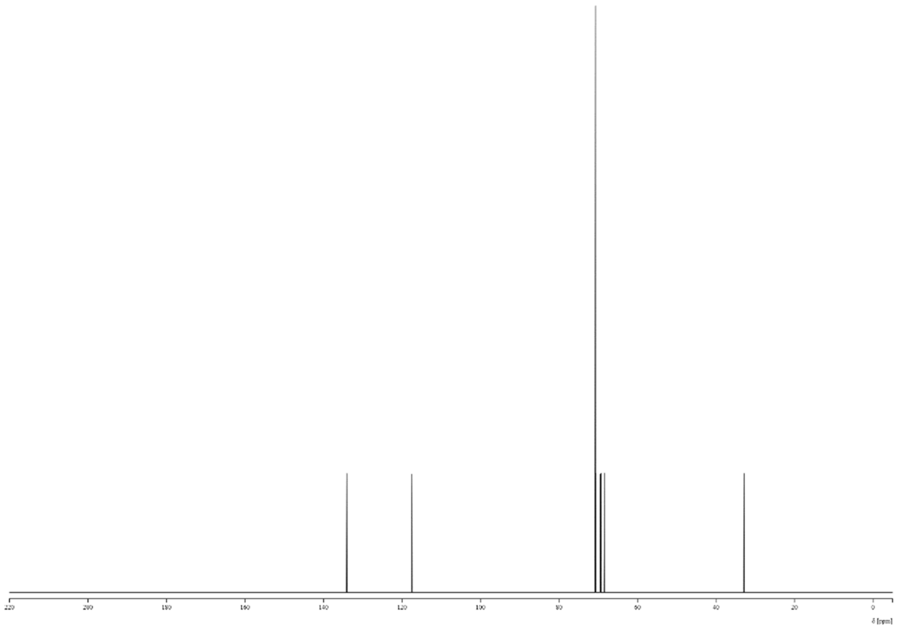

11) *O*-Phthalimido-tetraethylene glycol (**32)**

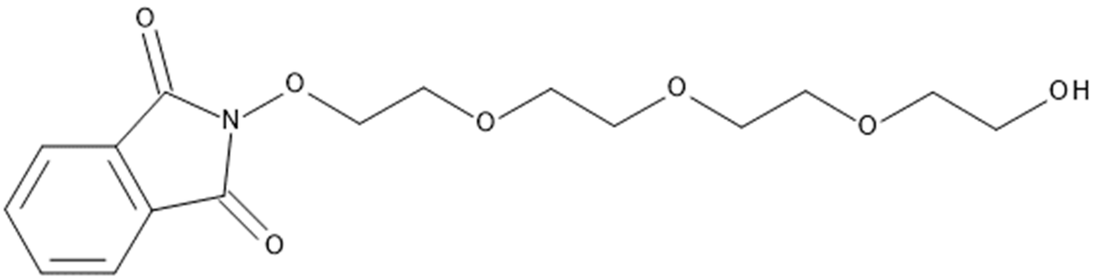

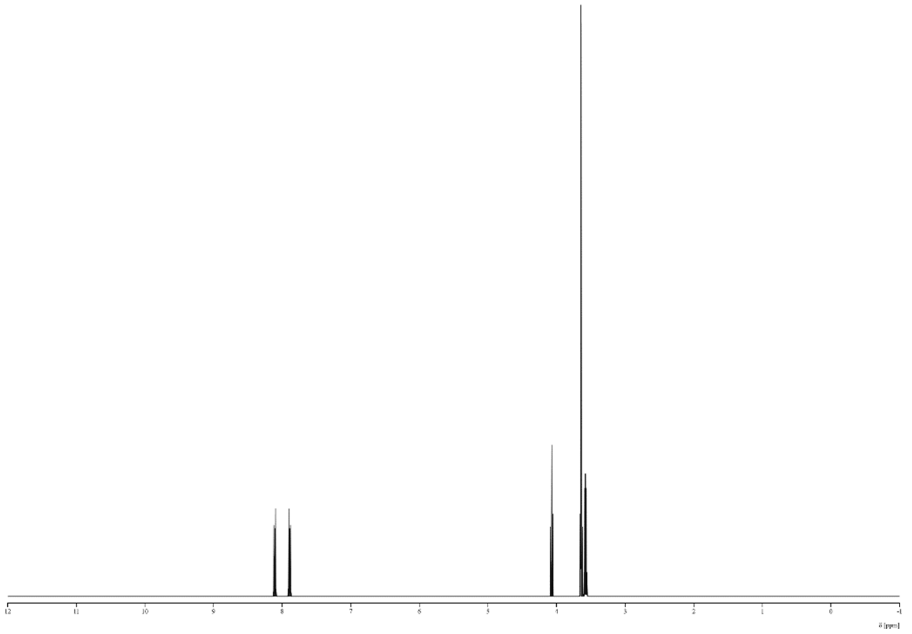

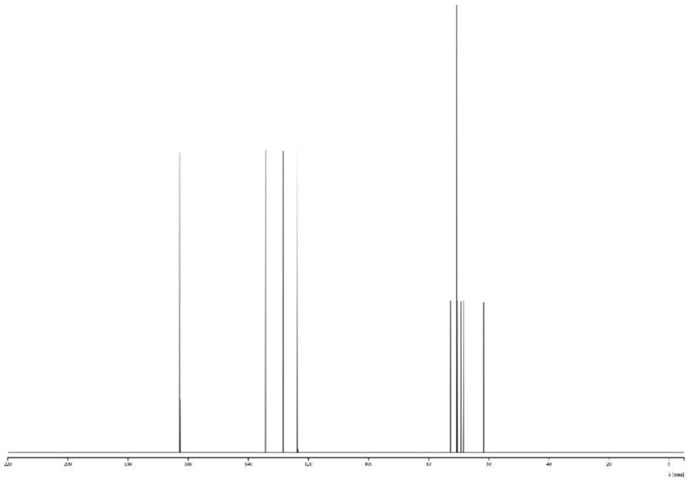

12) *O*-Phthalimido-(ω-thiotrityl)-tetraethylene glycol (**33**)

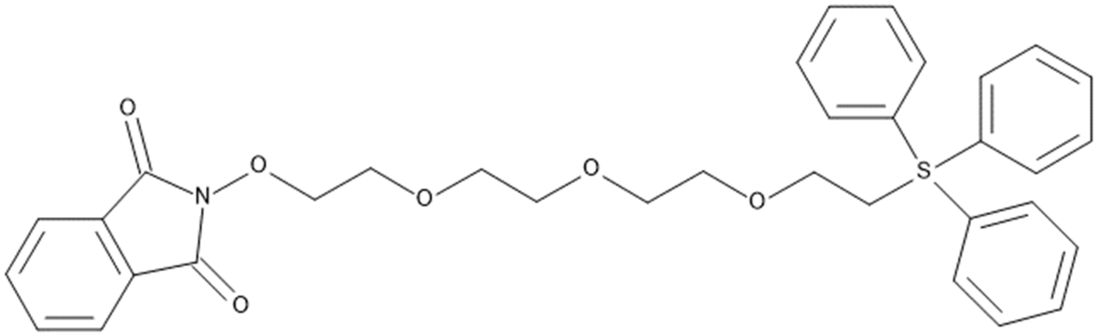

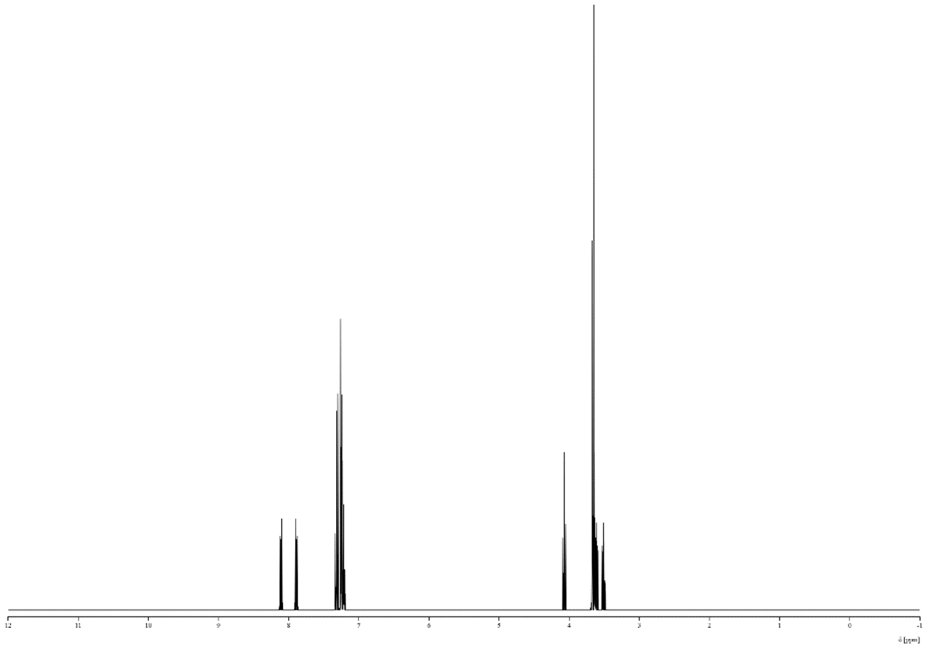

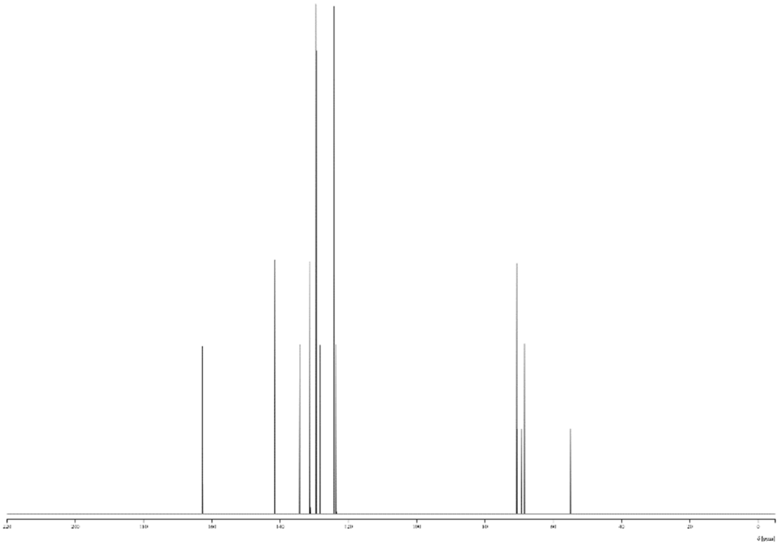

13) Hydroxylamino-(ω-thiotrityl)-tetraethylene-glycol (**34**)

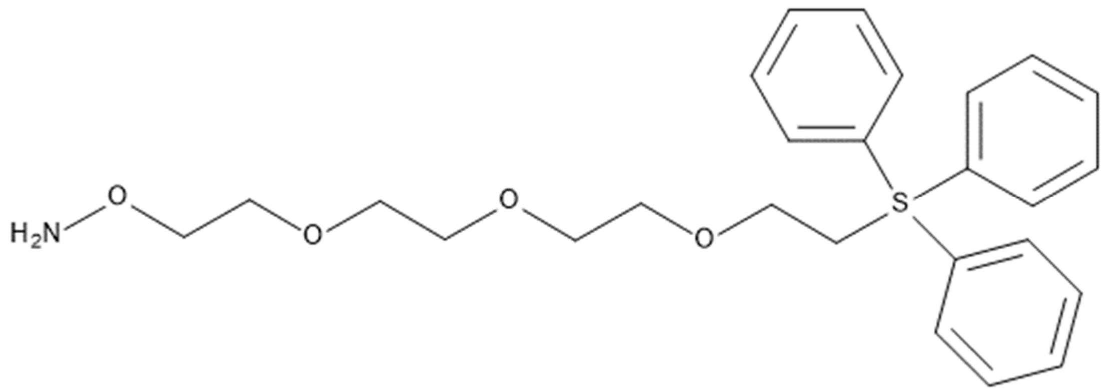

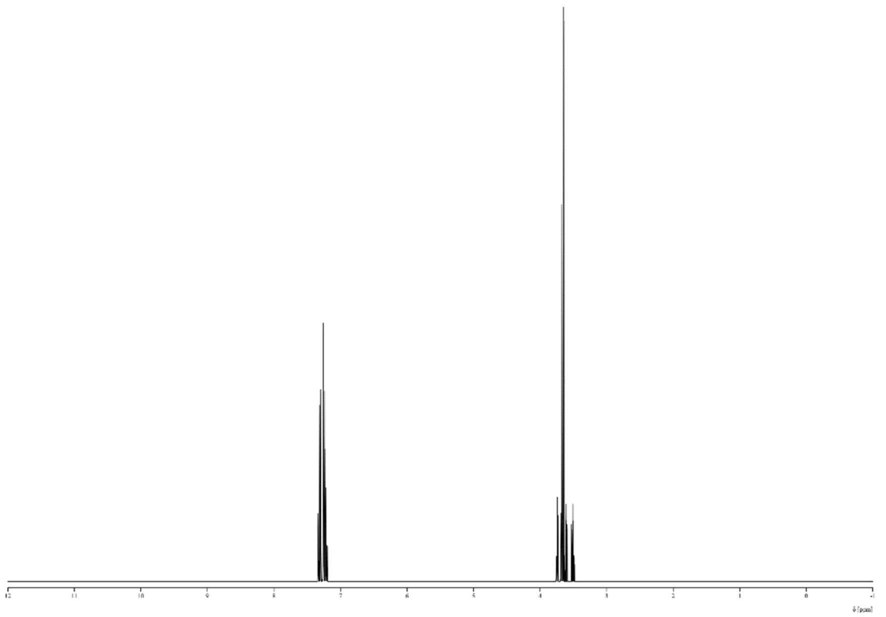

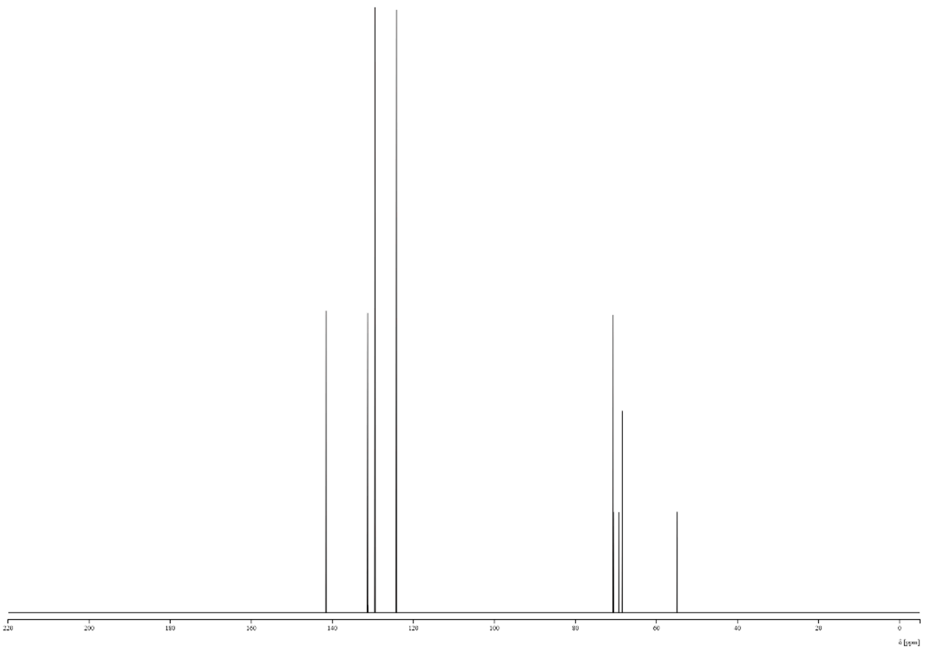

14) 4-[4-(ω-Thiotrityl)-tetraethylene-glycolyl-benzaldehyde-oxime]-butyl β-D-glucopyranoside (**35**)

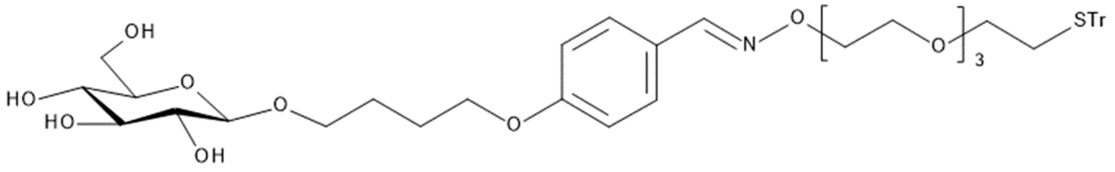

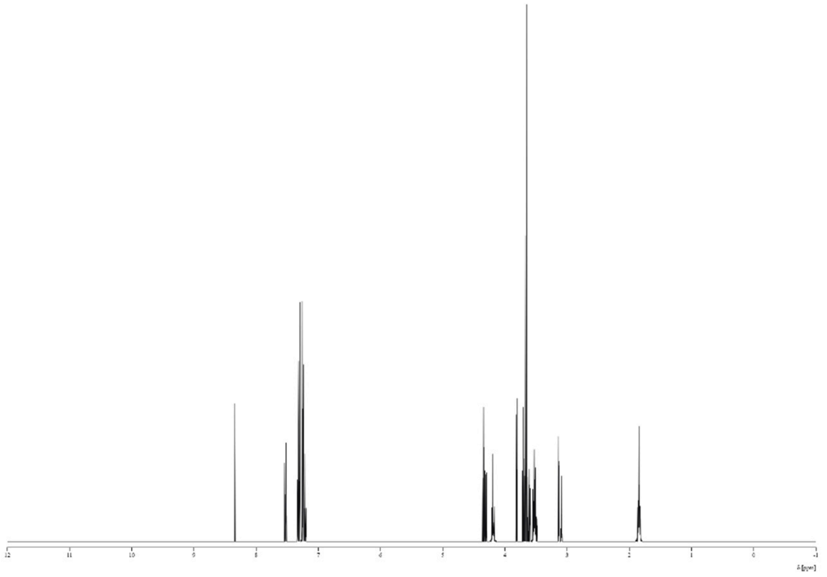

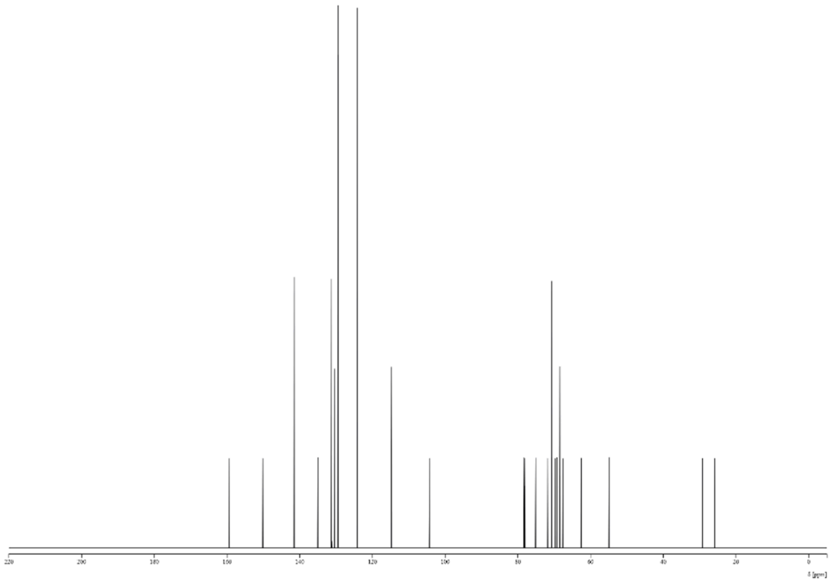

Supplement: Supplementary file 1 [file molecules-30-03765-s001.zip › molecules-3747481-supplementary.pdf]
